# Single‐Cell Profiling Reveals Clonally Expanded CX3CR1^+^ T Cells in Anti‐NMDA Receptor Encephalitis

**DOI:** 10.1002/advs.77608

**Published:** 2026-09-08

**Authors:** Lin Yan, Erdi Zhang, Yuwen Ma, Zirui Meng, Sisi Li, Yimeng Ren, Xiaohe Tian, Yangyi He, Giuseppe Battaglia, Dong Zhou, Yun Liao, Binwu Ying, Jinmei Li, Minjin Wang

**Affiliations:** ^1^ Department of Laboratory Medicine West China Hospital Sichuan University Chengdu Sichuan China; ^2^ Clinical Laboratory Medicine Research Center West China Hospital Sichuan University Chengdu Sichuan China; ^3^ Sichuan Clinical Research Center for Laboratory Medicine Chengdu Sichuan China; ^4^ Department of Laboratory Medicine West China Hospital of Stomatology Sichuan University Chengdu Sichuan China; ^5^ Department of Breast Cancer Center Chongqing University Cancer Hospital Chongqing China; ^6^ LKS Faculty of Medicine The University of Hong Kong Hong Kong China; ^7^ Functional and Molecular Imaging Key Laboratory of Sichuan Province Huaxi MR Research Center (HMRRC) Department of Radiology West China Hospital Sichuan University Chengdu Sichuan China; ^8^ Department of Surgery and Surgical Specializations University of Barcelona Barcelona Spain; ^9^ Institute for Bioengineering of Catalunya (IBEC) The Barcelona Institute of Science and Technology Barcelona Spain; ^10^ Department of Chemistry University College London (UCL) London UK; ^11^ Department of Neurology West China Hospital Sichuan University Chengdu Sichuan China; ^12^ Institution of Medical and Engineering Integration for Molecular Diagnosis West China Hospital Sichuan University Chengdu Sichuan China

**Keywords:** anti‐NMDA receptor encephalitis, CX3CR1, neuroinflammation, single‐Cell multi‐omics, T cell receptor clonality, T Cells

## Abstract

Anti‐*N‐methyl‐D‐aspartate* receptor encephalitis (NMDAR‐E) is a prevalent autoimmune neurological disorder, yet T cell‐driven immunopathology and its contributions to therapeutic resistance and fulminant neuroinflammation remain poorly defined. Integrative single‐cell multi‐omic profiling, combining single‐cell RNA sequencing, single‐cell T cell receptor (TCR) sequencing, and cellular indexing of transcriptomes and epitopes by sequencing, is applied to systematically map peripheral and cerebrospinal fluid (CSF) immune landscapes in treatment‐naïve NMDAR‐E patients, with orthogonal validation conducted across independent cohorts via multiparameter flow cytometry and ex vivo NR1 peptide stimulation. Clonally expanded CX3CR1‐expressing CD4^+^ and CD8^+^ T cells are identified as a core disease‐associated effector population, characterized by high predicted affinity for the pathogenic GluN1 N368/G369 epitope, robust proinflammatory and cytotoxic transcriptional programs, and enhanced intercellular crosstalk with activated B cells through major histocompatibility complex (MHC), CD99, and macrophage migration inhibitory factor signaling axes. Preferentially enriched in patient CSF, these cells produce elevated interferon‐γ (IFN‐γ) and tumor necrosis factor‐α (TNF‐α) upon antigen‐specific stimulation, collectively highlighting a disease‐associated inflammatory T cell state linked to peripheral immune activation and CNS immune remodeling, with potential translational relevance for future biomarker and therapeutic investigation.

## Graphical abstract

CX3CR1^+^ T cells are associated with peripheral and central immune alterations in anti‐NMDA receptor encephalitis (NMDAR‐E). They show potential responsiveness to GluN1 (NR1) peptides, potential interactions with peripheral B cells, clonal expansion, increased CD137/CD154 expression, and inflammatory cytokine production. Their preferential cerebrospinal fluid (CSF) enrichment and CX3CL1‐related features, together with plasma cell and anti‐NMDAR antibody responses, suggest coordinated involvement in neuroinflammation.

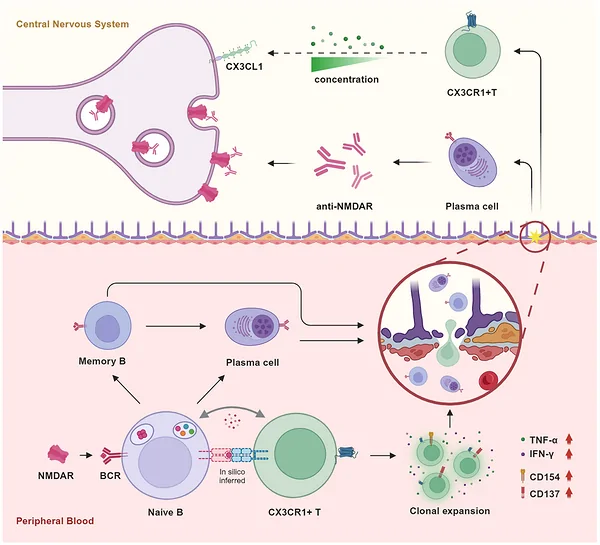

## 1 Introduction

Anti‐*N‐methyl‐D‐aspartate* receptor encephalitis (NMDAR‐E) is among the most prevalent and clinically consequential autoimmune neurological disorders. Over the past two decades, understanding of NMDAR‐E has evolved substantially, and the disease is now recognized as a well‐defined clinical entity [1, 2]. Substantial progress has concurrently been achieved in targeted immunotherapeutic interventions and symptom‐directed management approaches [3, 4]. Despite these advances, substantial challenges remain in the clinical management of NMDAR‐E. On the one hand, approximately 40% of patients show a limited or absent response to first‐line immunotherapy [5], and 25% experience disease relapse [6, 7]. On the other hand, a significant proportion of patients with severe disease develop systemic or central nervous system (CNS) hyperinflammatory states, which represent a major contributor to mortality [5, 8]. Although current clinical cohorts and experimental models have established the role of B cell antibody secretion, heterogeneity, and class switching in NMDAR‐E [9, 10, 11], this B‐cell‐centric paradigm may not fully explain the contribution of other immune compartments, thereby precluding a comprehensive elucidation of the mechanisms underlying immunotherapy resistance, drivers for disease relapse, and upstream triggers and downstream signaling cascades for fulminant neuroinflammation. These challenges underscore major gaps in our understanding of the immunopathological mechanisms underlying NMDAR‐E.

Current evidence supports a dual pathogenic role for T cells in autoimmune CNS disorders. On one hand, T cells can drive pathogenic antibody production by facilitating B cell activation, somatic hypermutation (SHM), and immunoglobulin class switching through three key mechanisms: recognition of antigen‐presenting cells (APCs), delivery of co‐stimulatory signals, and cytokine secretion [12, 13, 14]. On the other hand, activated autoreactive T cells polarize into proinflammatory subsets that directly infiltrate the CNS, exacerbating neuroinflammation [15, 16]. Consistent with this framework, single‐cell studies in NMDAR‐E suggest reciprocal interactions between T cells and B cells. B cells show enhanced antigen presentation through major histocompatibility complex (MHC) II, while signals from T follicular helper cells (Tfh) may in turn promote the differentiation of B cells into antibody‐secreting cells (ASCs), collectively sustaining autoreactive humoral immunity [17]. Beyond their interactions with B cells, T cells in the peripheral blood and cerebrospinal fluid (CSF) of patients with NMDAR‐E exhibit cytotoxic and inflammatory transcriptional programs [18]. Increased levels of inflammatory cytokines, including IFN‐γ, IL‐17, and TNF‐α, have also been reported in peripheral blood or CSF, further supporting the presence of systemic and intrathecal inflammation [19]. The involvement of T cells is further supported by several observations, including postmortem evidence of concurrent T and B cell infiltration in brain tissue and the complete absence of disease onset in murine models lacking mature T and B lymphocytes [19, 20, 21]. Collectively, these findings underscore T cells as pivotal orchestrators of both humoral autoimmunity and neuroinflammation in NMDAR‐E. Elucidating the interconnected roles of antigen‐specific T cell receptor (TCR) clonality, inflammatory effector function, and bidirectional T‐B cell crosstalk will help define how T cells contribute to sustained autoimmunity and pathogenic cytokine dysregulation, thereby advancing mechanistic understanding of the core immunopathological processes underlying this disease.

In this study, we integrated single‐cell RNA sequencing (scRNA‐seq), single‐cell T cell receptor sequencing (scTCR‐seq), and surface protein profiling by cellular indexing of transcriptomes and epitopes by sequencing (CITE‐seq) to systematically define disease‐associated T cell states in NMDAR‐E. These findings were further validated in an independent expanded cohort using multiparameter flow cytometry and an ex vivo NR1 peptide stimulation assay. This integrative approach identified antigen‐experienced T cell subsets with predicted TCR‐epitope interactions involving NMDAR‐associated peptides, revealed aberrant T‐B cell communication networks, and uncovered inflammatory effector programs associated with disease. Together, our findings provide new insight into the cellular and molecular immunopathogenesis of NMDAR‐E and provide a framework for future investigation of selective immunomodulatory strategies, particularly in treatment‐resistant disease.

## 2 Results

### 2.1 Single‐Cell Profiling Identifies Altered Immune Cell Proportions in NMDAR‐E

**FIGURE 1:**
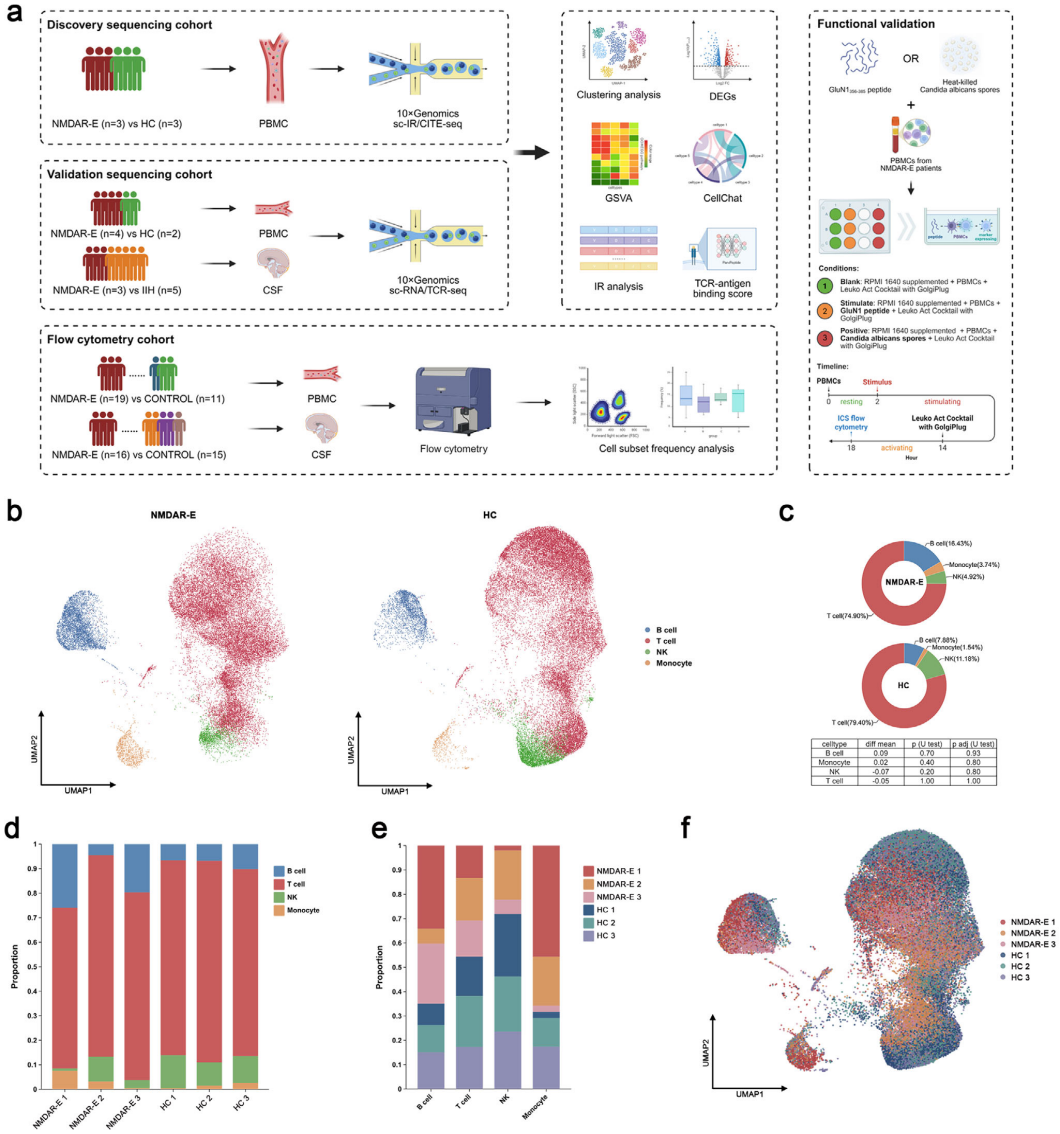
Study design and primary immune profiling of peripheral and central immune compartments in NMDAR‐E. (a) Schematic overview of the study design. In the discovery cohort, PBMCs from treatment‐naïve patients with NMDAR‐E (n = 3) and HC (n = 3) were profiled using 10x Genomics single‐cell immune repertoire sequencing with CITE‐seq (sc‐IR/CITE‐seq). In the validation sequencing cohort, PBMC samples (NMDAR‐E, n = 4; HC, n = 2) and CSF samples (NMDAR‐E, n = 3; IIH, n = 5) were analyzed using 10x Genomics single‐cell RNA and TCR sequencing (scRNA/TCR‐seq). Downstream analyses included clustering, differentially expressed gene (DEG) analysis, gene set variation analysis (GSVA), cell‐cell communication analysis (CellChat), immune repertoire profiling, and TCR‐antigen binding score prediction. An independent flow cytometry cohort comprising PBMC (NMDAR‐E, n = 19; HC, n = 11) and CSF (NMDAR‐E, n = 16; control, n = 15) samples was used for cell subset frequency analysis. Functional validation was performed by stimulating PBMCs from patients with NMDAR‐E using either the GluN1_356‐385_ peptide or heat‐killed *Candida albicans* (HKCA) spores, followed by intracellular cytokine staining (ICS) flow cytometry. (b) Uniform manifold approximation and projection (UMAP) projections of major immune cell populations identified from PBMCs in the discovery cohort, shown separately for NMDAR‐E and HC, including T cells, B cells, NK cells, and monocytes. All subsequent analyses (c–f) are based on PBMC data from the discovery cohort. (c) Donut charts showing the overall proportions of major immune cell populations in the NMDAR‐E and HC groups. (d) Stacked bar plots showing the relative composition of major immune cell populations in each individual sample. (e) Stacked bar plots showing the sample‐of‐origin composition within each major immune cell population. (f) UMAP projection of all PBMCs colored according to sample origin.

Single‐cell transcriptomic, immune repertoire, and surface protein profiling (scRNA‐seq, scTCR‐seq, and CITE‐seq) were conducted on peripheral blood mononuclear cells (PBMCs) from three treatment‐naïve patients with NMDAR‐E and three matched healthy controls (HCs), yielding a total of 59 507 high‐quality cells after quality control (Table S1 for sample details). To validate immune alterations, PBMCs from additional NMDAR‐E patients and HCs, and CSF samples from NMDAR‐E and idiopathic intracranial hypertension (IIH) patients, were subjected to parallel single‐cell profiling. Orthogonal validation was then performed in an independent cohort using multiparameter flow cytometry, followed by functional validation through ex vivo NR1 peptide stimulation of PBMCs (Figure 1a). Following integrated normalization, weighted nearest neighbor uniform manifold approximation and projection (wnnUMAP) was applied for clustering. Major immune populations were delineated and annotated based on canonical marker expression: T cells (CD3E, CD4, CD8A/B, and CD28), B cells (CD19, CD20, CD79A/B, and PAX5), natural killer (NK) cells (CD16, CD56, KLRD1, and KLRF1), and monocytes (CD14) (Figure 1b and Figure S1). Each major immune population was subsequently resolved into finer cellular subsets based on lineage‐specific transcriptional features (Figure S1). T cells constituted the predominant immune subset in both groups (74.9% in NMDAR‐E vs. 79.4% in HCs), whereas B cells (16.4% vs. 7.9%) and monocytes (3.7% vs. 1.5%) were notably enriched in the NMDAR‐E group, accompanied by a relative reduction in NK cells (4.9% vs. 11.2%) (Figure 1c). Sample‐wise stratification confirmed consistent trends of elevated B cell and monocyte proportions across most NMDAR‐E cases (Figure 1d–f), indicating a distinct pattern of peripheral immune remodeling with potential relevance to disease pathogenesis.

### 2.2 B Cell Subsets are Transcriptionally Activated and Enriched for Antigen Presentation Pathways

**FIGURE 2:**
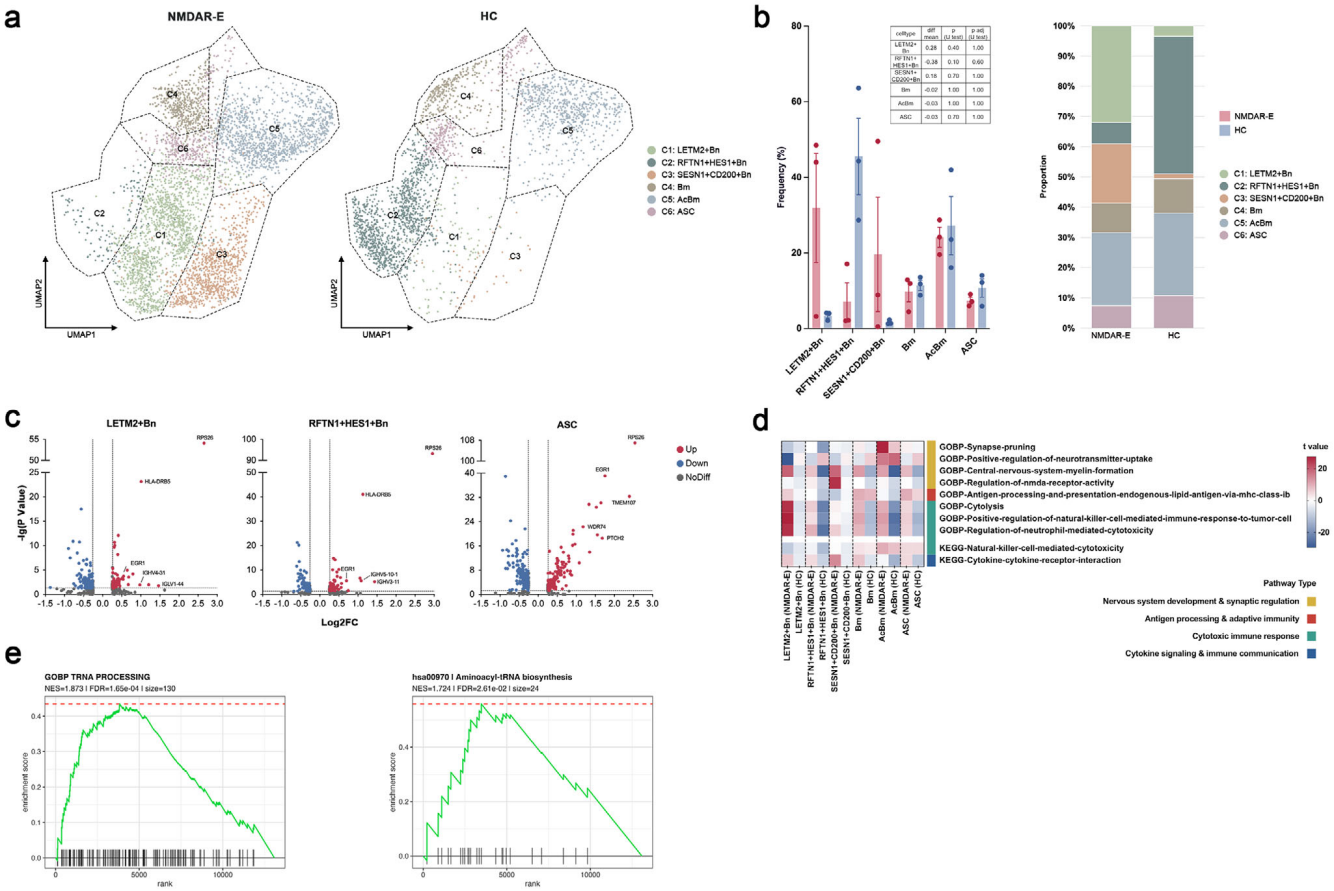
Secondary clustering and functional profiling of B cell subpopulations in NMDAR‐E. (a) UMAP projections of re‐clustered B cells from the NMDAR‐E (left) and HC (right) groups, identifying six transcriptionally distinct subpopulations annotated as LETM2^+^ naïve B cells (Bn) (C1), RFTN1^+^ HES1^+^ Bn (C2), SESN1^+^ CD200^+^ Bn (C3), memory B cells (Bm, C4), activated memory B cells (AcBm, C5), and ASC (C6). (b) Left, frequencies of each B cell subset in the NMDAR‐E and HC groups. Each dot represents one sample. Right, stacked bar plot showing the relative composition of B cell subsets within each group. (c) Volcano plots showing DEGs between NMDAR‐E and HC in representative B cell subsets (LETM2^+^ Bn, RFTN1^+^ HES1^+^ Bn, and ASC). Representative upregulated genes are highlighted. (d) GSVA heatmap showing representative significantly altered GOBP and KEGG pathways across B cell subsets. Among the significantly altered pathways (p < 0.05), representative pathways were selected for visualization based on their biological relevance to B cell function and NMDAR‐E. Heatmap colors represent t values. (e) Representative GSEA plots comparing all B cells from NMDAR‐E and HC, showing enrichment of the GOBP tRNA processing and KEGG aminoacyl‐tRNA biosynthesis pathways in NMDAR‐E.

Given the central role of B cells as direct producers of pathogenic autoantibodies in NMDAR‐E, we first conducted an in‐depth analysis. Unsupervised clustering of B cells identified six distinct subsets. Integration with cell annotation and pseudotime analysis (Figure S2) revealed three functionally distinct naive B cell clusters: RFTN1^+^ HES1^+^ (B cell receptor signaling suppression and transcriptional repression) predominated in controls, while LETM2^+^ (mitochondrial homeostasis) and SESN1^+^ CD200^+^ (oxidative stress and immunosuppression) B cell subsets were enriched in patients (Figure 2a,b). Patient‐derived B cells exhibited heightened transcriptional activity (e.g., RPS26, EGR1), accompanied by upregulation of MHC‐II (e.g., HLA‐DRB5) and B cell receptor (BCR) signaling genes (including IGHV/IGLV V‐genes such as IGHV4‐31, IGLV1‐44) (Figure 2c and Figure S2). Complementary gene set enrichment analysis (GSEA) identified significant enrichment of tRNA processing (Gene Ontology Biological Process, GOBP; NES = 1.87, FDR = 1.65 × 10^−4^) and aminoacyl‐tRNA biosynthesis (Kyoto Encyclopedia of Genes and Genomes, KEGG, hsa00970; NES = 1.72, FDR = 0.026) in patient B cells (Figure 2e), consistent with increased transcriptional and proliferative programs. GSVA revealed enrichment of genes associated with NMDA receptor regulation (go:2000310), synapse pruning (go:0098883), cytotoxicity (natural killer cell‐mediated cytotoxicity, hsa04650), and T cell interaction‐related pathways (antigen processing and presentation of endogenous lipid antigen via MHC class Ib, go:0048006) (Figure 2d), suggesting that B cells in these patients may play a novel role in enhanced antigen presentation and neuroinflammatory injury.

### 2.3 T Cell Heterogeneity Reflects Pro‐Inflammatory and Neurotoxic Potential in NMDAR‐E

**FIGURE 3:**
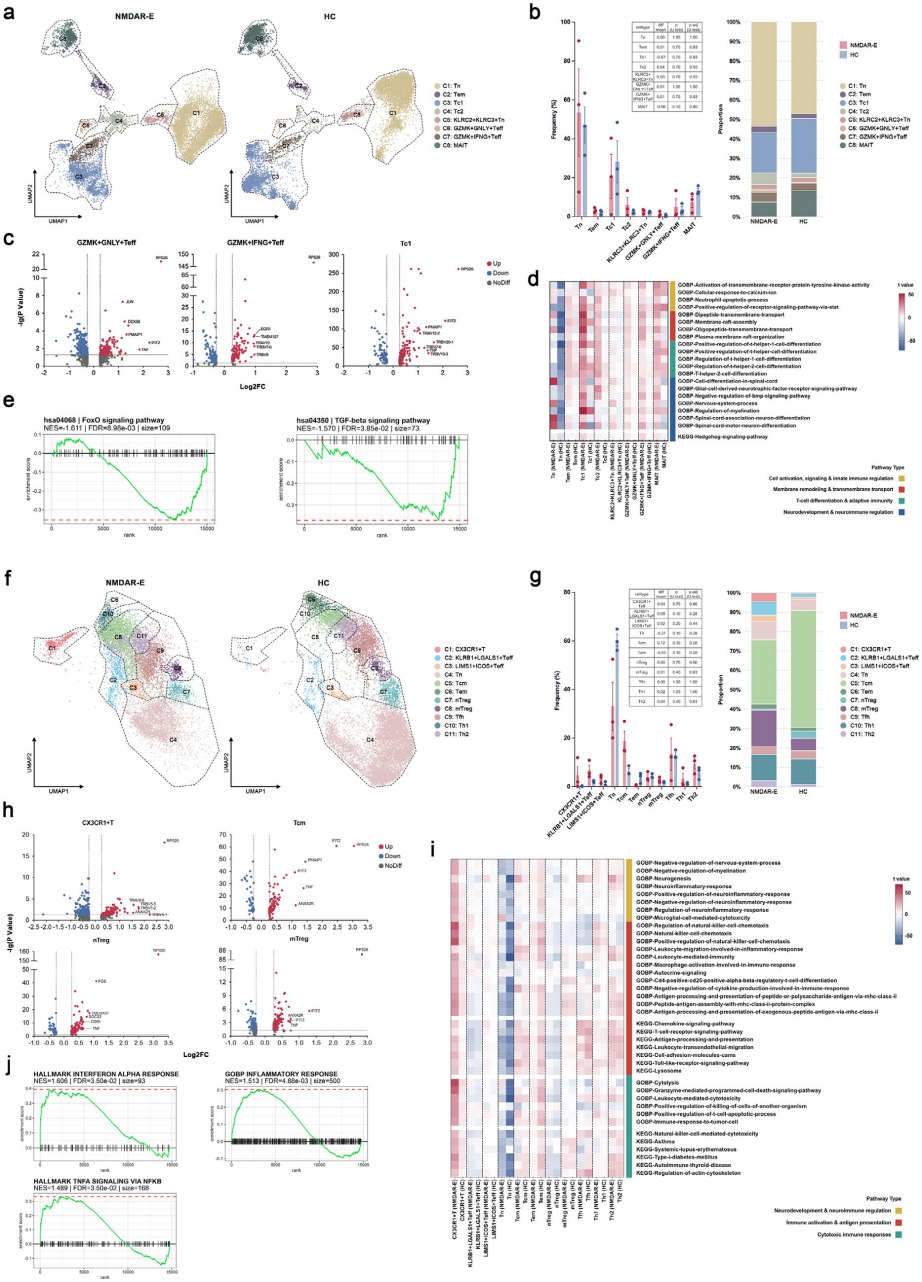
Secondary clustering and functional characterization of CD8^+^ and CD4^+^ T cell subsets in NMDAR‐E. (a) UMAP projections of re‐clustered CD8^+^ T cells from the NMDAR‐E (left) and HC (right) groups, identifying eight transcriptionally distinct subpopulations, including naïve T cells (Tn) (C1), effector memory T cells (Tem) (C2), type 1 cytotoxic T cells (Tc1) (C3), type 2 cytotoxic T cells (Tc2) (C4), KLRC2^+^ KLRC3^+^ Tn (C5), GZMK^+^ GNLY^+^ Teff (C6), GZMK^+^ IFNG^+^ Teff (C7), and mucosal‐associated invariant T cells (MAIT) (C8). (b) Left, frequencies of each CD8^+^ T cell subset in the NMDAR‐E and HC groups. Each dot represents one sample. Right, stacked bar plot showing the relative composition of CD8^+^ T cell subsets within each group. (c) Volcano plots showing DEGs between NMDAR‐E and HC in representative CD8^+^ T cell subsets (GZMK^+^ GNLY^+^ Teff, GZMK^+^ IFNG^+^ Teff, and Tc1). Representative upregulated genes are highlighted. (d) GSVA heatmap showing representative significantly altered GOBP and KEGG pathways across CD8^+^ T cell subsets. Among the significantly altered pathways (p < 0.05), representative pathways were selected for visualization based on their biological relevance to CD8^+^ T cell function and NMDAR‐E. Heatmap colors represent t values. (e) Representative GSEA plots comparing all CD8^+^ T cells from NMDAR‐E and HC, showing negative enrichment of the KEGG FoxO and TGF‐β signaling pathways in NMDAR‐E. (f) UMAP projections of re‐clustered CD4^+^ T cells from the NMDAR‐E (left) and HC (right) groups, identifying eleven transcriptionally distinct subpopulations, including CX3CR1^+^ T (C1), KLRB1^+^ LGALS1^+^ Teff (C2), LIMS1^+^ ICOS^+^ Teff (C3), Tn (C4), Tcm (C5), Tem (C6), nTreg (C7), mTreg (C8), Tfh (C9), type 1 helper T cells (Th1) (C10), and type 2 helper T cells (Th2) (C11). (g) Left, frequencies of each CD4^+^ T cell subset in the NMDAR‐E and HC groups. Each dot represents one sample. Right, stacked bar plot showing the relative composition of CD4^+^ T cell subsets within each group. (h) Volcano plots showing DEGs between NMDAR‐E and HC in representative CD4^+^ T cell subsets (CX3CR1^+^ T, Tcm, nTreg, and mTreg). Representative upregulated genes are highlighted. (i) GSVA heatmap showing representative significantly altered GOBP and KEGG pathways across CD4^+^ T cell subsets. Among the significantly altered pathways (p < 0.05), representative pathways were selected for visualization based on their biological relevance to CD4^+^ T cell function and NMDAR‐E. Heatmap colors represent t values. (j) Representative GSEA plots comparing all CD4^+^ T cells from NMDAR‐E and HC, showing enrichment of the Hallmark Interferon Alpha Response, GOBP Inflammatory Response, and Hallmark TNFA Signaling via NF‐κB gene sets in NMDAR‐E.

The phenotypic heterogeneity and transcriptional states of T cells were profiled to evaluate their contribution to neuroinflammatory immune activation and tissue injury. Within CD8^+^ T cells, several inflammatory effector subsets were identified, including effector T cells (Teff), such as GZMK^+^ GNLY^+^ Teff and GZMK^+^ IFNG^+^ Teff (Figure 3a,b and Figure S3), characterized by a receptor‐proximal TCR‐activation signature (e.g., JUN, EGR1, FOS) together with biased TRAV/TRBV usage (e.g., TRAV19, TRBV7‐6) (Figure 3c and Figure S3), consistent with TCR repertoire skewing associated with effector expansion. GSEA of a preranked gene list revealed significant negative enrichment of FoxO signaling (KEGG hsa04068; NES = −1.61, FDR = 0.009) and TGF‐β signaling (KEGG hsa04350; NES = −1.57, FDR = 0.038) in patient CD8^+^ T cells (Figure 3e), supporting MAPK pathway activation and organelle quality control through autophagy and mitophagy in effector activation. Functional enrichment highlighted pathways related to membrane signaling (go:0035442, go:0035672, go:0044857, go:0001765), T helper cell differentiation (go:0045627, go:0045628, go:0045064, go:0045625), and neurodevelopment (go:0050877, go:0031641, Hedgehog signaling: hsa04340), indicating that these CD8^+^ T cells exhibit marked immune activation and effector differentiation (Figure 3d). These subsets may participate in disease progression through clonal expansion and enhanced cytotoxic and inflammatory activity.

In parallel, CD4^+^ T cells also exhibited pronounced heterogeneity and disease‐associated shifts. Eleven subsets were delineated, including significantly expanded CX3CR1^+^ T cells and central memory T cells (Tcm) in NMDAR‐E patients (Figure 3f,g and Figure S4). Transcriptomic profiling revealed widespread upregulation of IFN/TNF‐related genes (e.g., IFIT2/3, ANXA2R, TNF), activation of metabolic and proliferative signatures (e.g., RPS26, FOS, MYC), and skewed TRAV/TRBV usage (e.g., TRAV8‐6, TRBV5‐5, TRBV7‐2), particularly in CX3CR1^+^ T cells (Figure 3h and Figure S4). GSEA of a preranked gene list showed positive enrichment of the Hallmark Interferon Alpha Response, GOBP Inflammatory Response, and Hallmark TNFA Signaling via NF‐κB gene sets, consistent with the direction of the DEG signatures (Figure 3j). GSVA across T cell subsets corroborated these signals, revealing enrichment of chemotactic (go:2000501, go:0035747, go:2000503, go:0002523, hsa04062), antigen presentation (go:0002504, go:0025030, go:0019886, hsa04612), neuroinflammatory (go:0150076, go:0150078), cytotoxic (go:0001909, go:0090634, hsa04650), and autoimmune‐related pathways (hsa05322, hsa05310, hsa04940, hsa05320) in NMDAR‐E patients. In contrast, regulatory T cells (Treg) displayed impaired migratory potential and possibly reduced suppressive function in NMDAR‐E patients (Figure 3i).

Collectively, these findings suggest that marked heterogeneity, clonal expansion, and inflammatory activation of CD4^+^ and CD8^+^ T cells are prominent features of NMDAR‐E and may contribute to a highly inflamed and dysregulated CNS immune milieu.

### 2.4 Multimodal Single‐Cell Characterization of the Inflammatory Phenotype and Crosstalk Potential of the CX3CR1^+^ T Cell Subset

**FIGURE 4:**
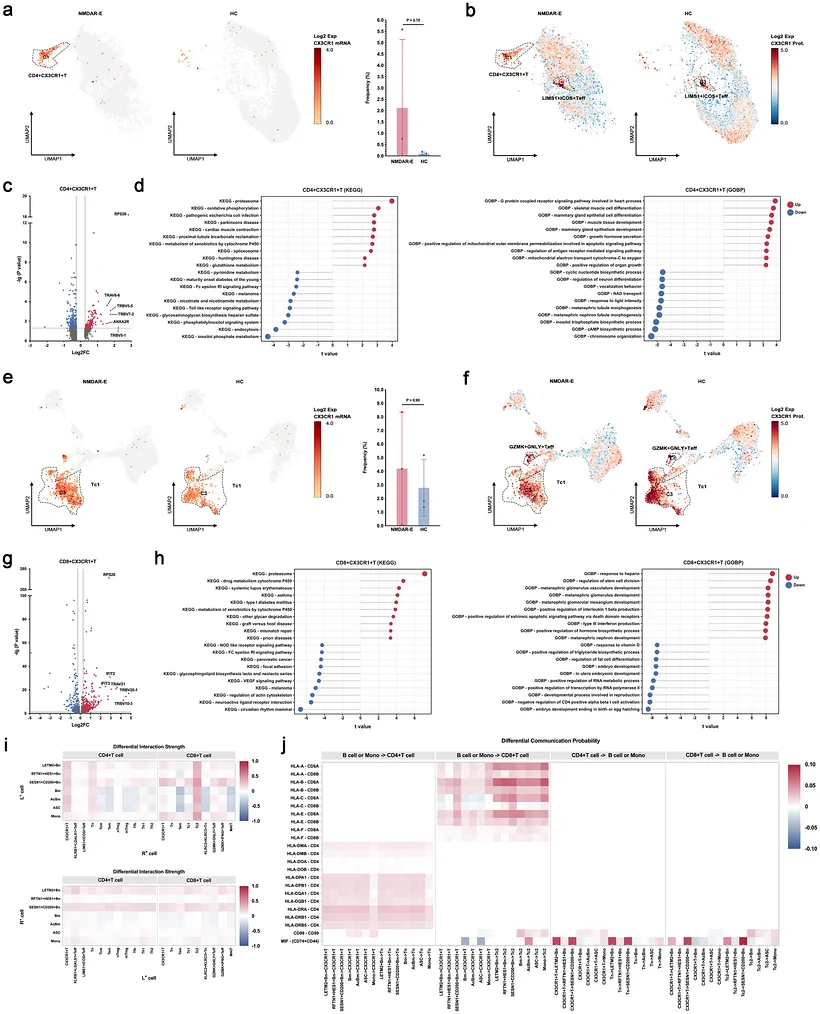
Transcriptomic, proteomic, and intercellular communication analyses of CX3CR1^+^ T cell subsets in NMDAR‐E. (a) UMAP projections showing CX3CR1 mRNA expression in CD4^+^ T cells from the NMDAR‐E (left) and HC (middle) groups. CX3CR1 expression is predominantly enriched in the CD4^+^ CX3CR1^+^ T subset. Right, frequencies of CD4^+^ CX3CR1^+^ T cells in the NMDAR‐E and HC groups. (b) UMAP projections showing surface CX3CR1 protein expression (CITE‐seq) in CD4^+^ T cells from the NMDAR‐E (left) and HC (right) groups. CX3CR1 protein is predominantly enriched in CD4^+^ CX3CR1^+^ T and LIMS1^+^ ICOS^+^ Teff cells. (c) Volcano plot showing DEGs between NMDAR‐E and HC in CD4^+^ CX3CR1^+^ T cells. Representative upregulated genes are highlighted. (d) GSVA dot plots showing representative significantly altered KEGG pathways (left) and GOBP terms (right) in CD4^+^ CX3CR1^+^ T cells. Among the pathways with p < 0.05, representative pathways were selected for visualization based on their biological relevance to T cell function and NMDAR‐E. (e) UMAP projections showing CX3CR1 mRNA expression in CD8^+^ T cells from the NMDAR‐E (left) and HC (middle) groups. CX3CR1 expression is predominantly enriched in the Tc1 subset. Right, frequencies of CD8^+^ CX3CR1^+^ T cells in the NMDAR‐E and HC groups. (f) UMAP projections showing surface CX3CR1 protein expression (CITE‐seq) in CD8^+^ T cells from the NMDAR‐E (left) and HC (right) groups. CX3CR1 protein is predominantly enriched in Tc1 and GZMK^+^ GNLY^+^ Teff cells. (g) Volcano plot showing DEGs between NMDAR‐E and HC in CD8^+^ CX3CR1^+^ T cells. Representative upregulated genes are highlighted. (h) GSVA dot plots showing representative significantly altered KEGG pathways (left) and GOBP terms (right) in CD8^+^ CX3CR1^+^ T cells. Among the pathways with p < 0.05, representative pathways were selected for visualization based on their biological relevance to T cell function and NMDAR‐E. (i) Heatmaps showing differential interaction strength inferred by CellChat (NMDAR‐E minus HC) between B cell subsets and CD4^+^ or CD8^+^ T cell subsets. Upper panel, ligand‐to‐receptor (L→R) interactions; lower panel, receptor‐to‐ligand (R→L) interactions. (j) Heatmaps showing differential communication probabilities (NMDAR‐E minus HC) for representative ligand‐receptor interactions between B cell or monocyte subsets and CD4^+^ or CD8^+^ T cell subsets, as inferred by CellChat.

A systematic comparison of all T cell subsets identified CD4^+^ CX3CR1^+^ T cells as those with the most prominent disease‐associated features in patients. Together with earlier reports that CX3CR1^+^ T cells can migrate into the CNS and exert effector functions [22], this led us to focus on this subset for detailed single‐cell analysis integrating transcriptomic and surface‐protein data. We therefore quantified CX3CR1 transcript levels in CD4^+^ and CD8^+^ effector T cells and used CITE‐seq to profile their surface protein expression, with the aim of delineating their functional states and intercellular communication properties in NMDAR‐E. At the mRNA level, in the CD4^+^ T cell compartment, the frequency of the CD4^+^ CX3CR1^+^ T subset was increased in patients (Figure 4a). In the CD8^+^ T cell compartment, CX3CR1^+^ Tc1 cells displayed a clearly distinct clustering pattern between patients and controls, with a higher abundance in patients (Figure 4e). At the protein level, CITE‐seq analysis corroborated the mRNA results (Figure 4b,f). DEGs revealed upregulation of TCR‐related genes in both CX3CR1^+^ T subsets, with immune activation‐related genes (e.g., ANXA2R) elevated in CD4^+^ T cells and interferon‐stimulated genes (e.g., IFIT2, IFIT3) increased in CD8^+^ T cells (Figure 4c,g). GSEA showed that CD4^+^ CX3CR1^+^ T cells were enriched for the inflammatory response and TNF‐α signaling via NF‐κB, whereas CD8^+^ CX3CR1^+^ T cells were enriched for cytokine‐cytokine receptor interaction, organic acid catabolism, and viral protein interaction with cytokine and cytokine receptor pathways (Figure S5). These pathway‐level enrichments are concordant with the DEG signatures and indicate an activated immune state in both subsets. Functional enrichment analyses indicated sustained activation of metabolic and proteolytic programs, along with neuroinflammation‐ and cytotoxicity‐associated pathways in both subsets (Figure 4d,h). To further characterize the functional state of CX3CR1^+^ T cells, we compared effector, activation, and exhaustion signatures between CX3CR1^+^ and CX3CR1^−^ T cells. Both CD4^+^ and CD8^+^ CX3CR1^+^ T cells exhibited markedly higher effector scores, whereas exhaustion scores were only modestly increased, indicating that these cells predominantly retained an activated effector state rather than a terminally exhausted phenotype (Figure S5).

CellChat analysis further revealed enhanced intercellular communication between CD4^+^ CX3CR1^+^ T cells, CD8^+^ CX3CR1^+^ T cells, and B cells, particularly naïve‐like subsets such as LETM2^+^ Bn and SESN1^+^ CD200^+^ Bn, as well as monocytes (Figure 4i). These interactions were primarily mediated through the MHC‐II‐CD4, MHC‐I‐CD8, CD99‐CD99, and macrophage migration inhibitory factor (MIF)‐(CD74 + CD44) signaling axes (Figure 4j), suggesting that CX3CR1^+^ T cells might contribute to antigen‐specific T‐B cell cooperation and amplification of inflammation in the context of NMDAR‐E.

### 2.5 CX3CR1^+^ T Cells Exhibit Clonality and NMDAR Epitope Recognition

**FIGURE 5:**
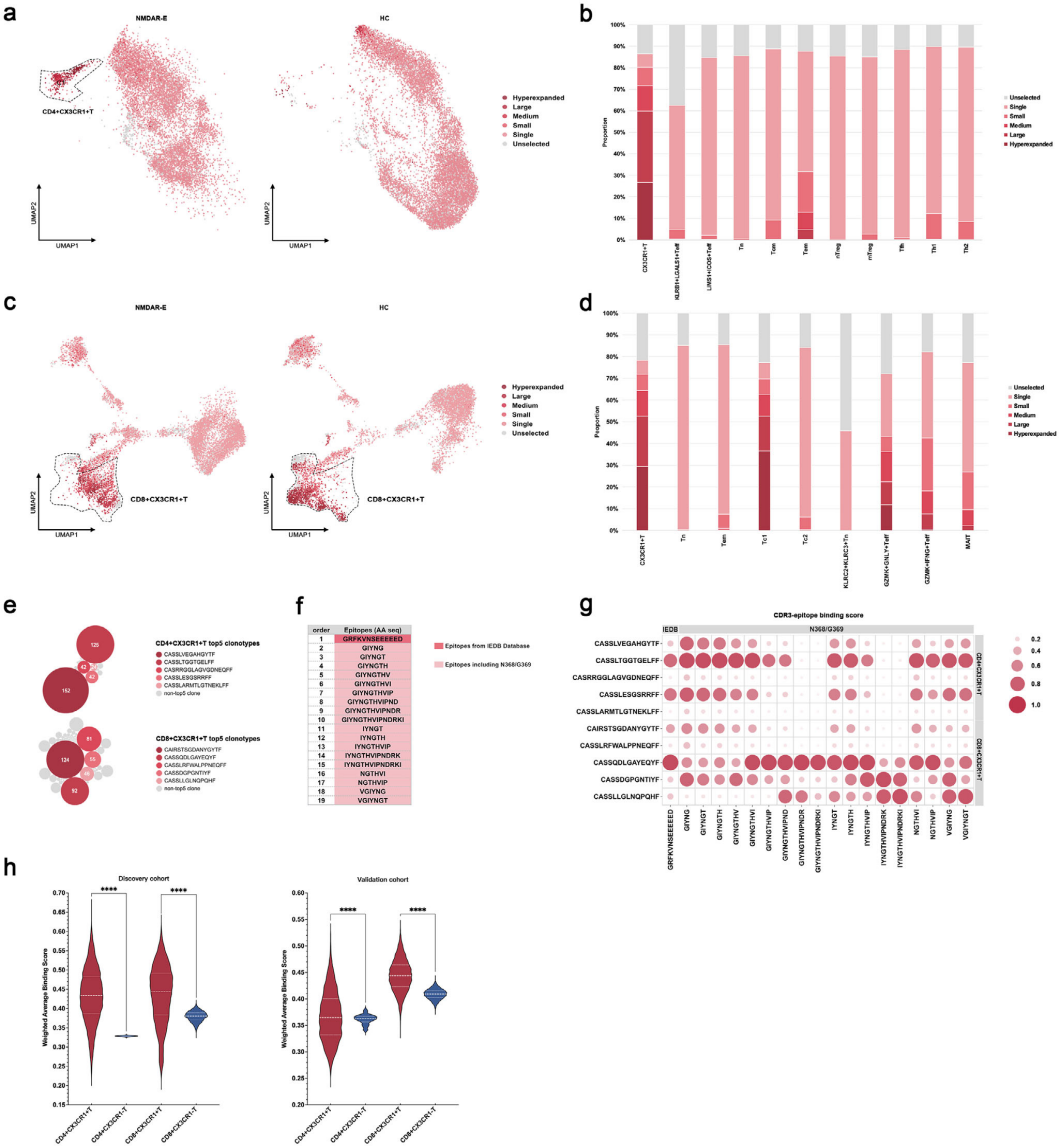
Clonal expansion and epitope binding prediction of CX3CR1^+^ T cells in NMDAR‐E. (a) UMAP projections of CD4^+^ T cells colored according to clonal expansion status. Clonal expansion was classified into six categories based on clone size: hyperexpanded (clone size ≥100), large (20–99), medium (5–19), small (2–4), single (1), and unselected (no TCR detected). (b) Relative proportions of cells with different clonal expansion levels across CD4^+^ T cell subsets. (c) UMAP projections of CD8^+^ T cells colored according to clonal expansion status. (d) Relative proportions of cells with different clonal expansion levels across CD8^+^ T cell subsets. (e) The top five expanded clonotypes identified in CD4^+^ CX3CR1^+^ T cells and CD8^+^ CX3CR1^+^ T cells. Circle size represents clonotype abundance, and CDR3 amino acid sequences are shown. (f) Candidate linear epitopes derived from the NR1 subunit. Epitopes include experimentally curated peptides from the IEDB database and all 5–15 amino acid peptides spanning the known antibody‐binding region containing residues N368/G369. Only epitopes predicted to have binding scores ≥0.85 with at least one of the top clonotypes are shown. (g) Predicted binding scores between CDR3 sequences from the top expanded clonotypes and NR1‐derived epitopes using PanPep. Dot size reflects the predicted binding score. (h) Weighted average predicted binding scores of NR1‐derived epitopes in CX3CR1^+^ and CX3CR1^−^ CD4^+^ and CD8^+^ T cells in the discovery and validation cohorts. Weighted averages were calculated using clonotype size as the weighting factor.

To further investigate the disease‐associated clonal features of CX3CR1^+^ T cells, TCR repertoire analysis revealed distinct clonal expansion patterns. Global TCR repertoire diversity was first evaluated using multiple complementary metrics. Overall diversity was comparable between NMDAR‐E and HC samples, and hyperexpanded clonotypes were detected in both groups (Figure S6). Visualization of clonotype distribution on the UMAP revealed marked clonal expansion of CX3CR1^+^ CD4^+^ T cells in patients, with hyperexpanded clonotypes predominating within this population (Figure 5a,b). CD8^+^ CX3CR1^+^ T cells similarly demonstrated clonal dominance, harboring hyperexpanded CD8^+^ T cell clonotypes (Figure 5c,d). Notably, we next compared the transcriptional states of expanded CD4^+^ and CD8^+^ CX3CR1^+^ T cells between NMDAR‐E and HC samples. Expanded T cells from patients exhibited enhanced type I interferon and cytotoxic effector programs, together with enrichment of lymphocyte activation, antigen‐receptor signaling, and leukocyte adhesion/trafficking pathways (Figures S7 and S8). These results indicate that the principal disease‐associated difference lies in the functional state of expanded T cells rather than in clonal expansion alone.

To explore potential interactions between TCRs and epitopes in NMDAR‐E, analysis of the TCR repertoires revealed features of clonally expanded CD4^+^ CX3CR1^+^ and CD8^+^ CX3CR1^+^ effector T cells. Multiple expanded clonotypes were identified in both subsets, including dominant complementarity‐determining region 3 (CDR3) sequences such as CASSLTGGTGELFF, CASSLRFWAIPNQEFF, and CASSQLGLNQPHF (Figure 5e). Top TCR clonotypes from both CD4^+^ CX3CR1^+^ T and CD8^+^ CX3CR1^+^ T subsets showed relatively high predicted TCR‐epitope interaction scores (score ≥ 0.85) to 19 GluN1 epitopes, of which 18 included the pathogenic N368/G369 region. At the same time, one was sourced from Immune Epitope Database (IEDB) and did not include N368/G369 (Figure 5f,g). Further comparison revealed that CX3CR1^+^ T cells displayed significantly higher average predicted binding scores than their CX3CR1^−^ counterparts within both the CD4^+^ and CD8^+^ memory and effector T cell compartments. This difference was reproduced in an independent validation cohort (Figure 5h). In contrast, peripheral B cell subsets showed minimal clonal expansion, with intergroup clonotype differences attributable to differences in cell counts (Figure S8). These computational findings identify CX3CR1^+^ T cell clonotypes with predicted interactions with GluN1 derived epitopes and provide hypothesis generating evidence for further investigation of their antigenic specificity.

### 2.6 Validated Expansion and Inflammatory Activation of CX3CR1^+^ T Cells in Peripheral and CNS Compartments

**FIGURE 6:**
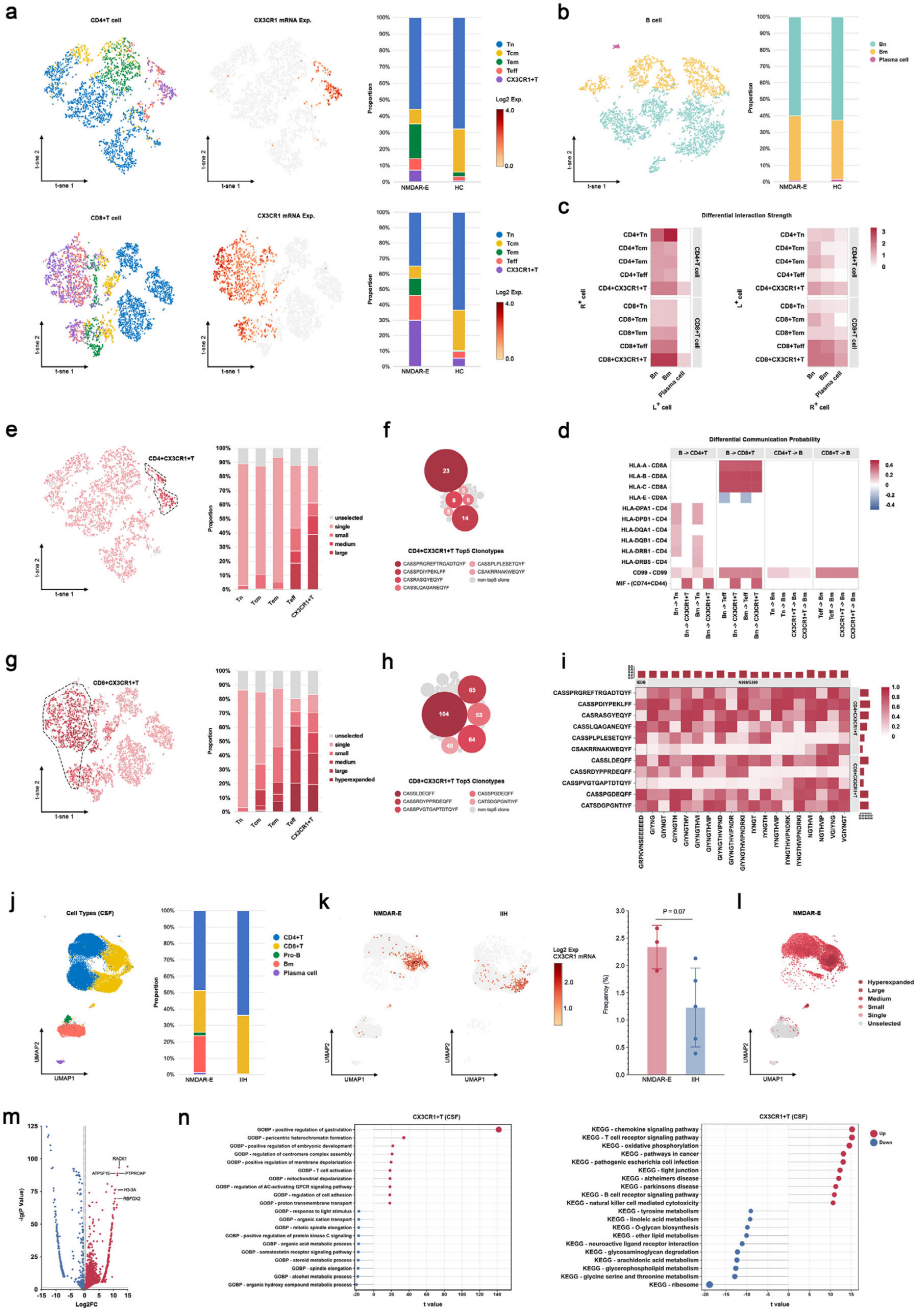
Validation of CX3CR1^+^ T cell features in independent PBMC and CSF datasets. (a) Validation of PBMC T cell subsets. Left, t‐SNE projections showing annotated CD4^+^ and CD8^+^ T cell subsets. Middle, CX3CR1 mRNA expression. Right, relative proportions of T cell subsets in the NMDAR‐E and HC groups. Legends corresponding to the left, middle, and right panels are displayed on the far right. (b) Validation of PBMC B cell subsets. Left, t‐SNE projection showing annotated B cell populations, including Bn, Bm, and plasma cells. Right, relative proportions of B cell subsets in the NMDAR‐E and HC groups. (c) Heatmaps showing differential interaction strength inferred by CellChat (NMDAR‐E minus HC) between B cell and T cell subsets. Left, ligand‐to‐receptor (L→R) interactions; right, receptor‐to‐ligand (R→L) interactions. (d) Heatmap showing differential communication probabilities (NMDAR‐E minus HC) for representative ligand‐receptor interactions between B cell subsets and selected CD4^+^ and CD8^+^ T cell subsets. (e) Clonal expansion analysis of CD4^+^ T cells. Left, t‐SNE projection colored according to clonal expansion status. Right, relative proportions of different clonal expansion levels across CD4^+^ T cell subsets. CD4^+^ CX3CR1^+^ T cells exhibited marked clonal expansion. (f) Top five hyperexpanded clonotypes identified in CD4^+^ CX3CR1^+^ T cells (six clonotypes are shown because of a tie). Circle size represents clonotype abundance, and labels indicate CDR3 amino acid sequences. (g) Clonal expansion analysis of CD8^+^ T cells. Left, t‐SNE projection colored according to clonal expansion status. Right, relative proportions of different clonal expansion levels across CD8^+^ T cell subsets. CD8^+^ CX3CR1^+^ T cells exhibited marked clonal expansion. (h) The top five hyperexpanded clonotypes identified in CD8^+^ CX3CR1^+^ T cells. (i) Heatmap showing predicted binding scores between CDR3 sequences from the top expanded clonotypes identified in CD4^+^ CX3CR1^+^ T and CD8^+^ CX3CR1^+^ T cells (11 clonotypes in total) and the candidate NR1‐derived linear epitopes shown in Figure 5f. Heatmap colors represent predicted binding scores. The bar plots above and to the right of the heatmap indicate the mean predicted binding scores for each column and row, respectively. (j) Validation of the CSF dataset. Left, UMAP projection showing annotated immune cell populations. Right, relative proportions of immune cell populations in the NMDAR‐E and IIH groups. (k) UMAP projections showing CX3CR1 mRNA expression in CSF‐derived T cells from the NMDAR‐E (left) and IIH (middle) groups. CX3CR1 expression is predominantly enriched in the CD8^+^ T cell subset. Right, frequencies of CX3CR1‐expressing CD8^+^ T cells in the NMDAR‐E and IIH groups. (l) UMAP projection of CSF‐derived T cells from the NMDAR‐E group colored according to clonal expansion status. (m) Volcano plot showing DEGs between NMDAR‐E and IIH in CX3CR1‐expressing CD8^+^ T cells from CSF. (n) GSVA dot plots showing representative significantly altered GOBP terms (left) and KEGG pathways (right) in CX3CR1‐expressing CD8^+^ T cells from CSF.

To further validate the disease‐associated expansion and inflammatory phenotype of CX3CR1^+^ T cells in NMDAR‐E, an independent single‐cell dataset from peripheral blood and CSF was examined. The cohort included patients with NMDAR‐E, HCs, and individuals with IIH (see Table S2 for details). In peripheral samples, CX3CR1 expression remained enriched in CD4^+^ and CD8^+^ T subsets in patients, while B cells composed both naïve‐like and memory subsets without major shifts in overall distribution (Figure 6a,b). In the validation cohort, CX3CR1^+^ T cells likewise showed DEG, GSEA, and GSVA results concordant with the immune‐activation trends observed in the discovery cohort (Figure S7). CellChat analysis revealed enhanced ligand‐receptor interactions between patient‐derived CX3CR1^+^ CD4^+^/CD8^+^ T cells and naïve/memory B cells, primarily mediated via MHC‐II‐CD4, MHC‐I‐CD8, CD99‐CD99, and MIF‐(CD74 + CD44) signaling axes (Figure 6c,d). Clonotype analysis further confirmed significant clonal expansion (Figure 6e,g), and TCR repertoire profiling revealed dominant clonotypes with high predicted binding scores to the pathogenic GluN1 N368/G369 epitope of the NMDAR (Figure 6f,h,i). In CSF samples from NMDAR‐E patients, CX3CR1^+^ T cells remained increased and formed a more distinct cluster compared with controls (Figure 6j,k). Consistently, five‐tier TCR clonal expansion mapping in CSF showed that CX3CR1^+^ T cells co‐localized with highly expanded clonotypes (Figure 6l). These cells exhibited elevated expression of transcriptional markers such as PTPRCAP, ATP5F1E, and RACK1, consistent with increased activation and functional reprogramming, further supporting an activated inflammatory effector state of CX3CR1^+^ T cells in NMDAR‐E (Figure 6m). Consistent with this, CSF GSEA highlighted enrichment of B cell activation, T cell proliferation and activation, adaptive immune responses, antigen receptor‐mediated signaling, and leukocyte adhesion pathways (Figure S7); GSVA performed specifically on CSF CX3CR1^+^ T cells likewise demonstrated heightened activation, proliferation, adhesion, and antigen‐presentation programs, reinforcing an activated immune milieu. Pathway enrichment analysis indicated robust activation of chemokine signaling, immune effector function, metabolic processes, and neurological disease pathways (Figure 6n). These results collectively validate the inflammatory phenotype and central enrichment of CX3CR1^+^ T cells in an independent cohort, further supporting their role as a prominent inflammatory effector population in NMDAR‐E.

### 2.7 Multi‐Level Validation of CX3CR1^+^ T Cell Expansion and Functional Relevance in NMDAR‐E

**FIGURE 7:**
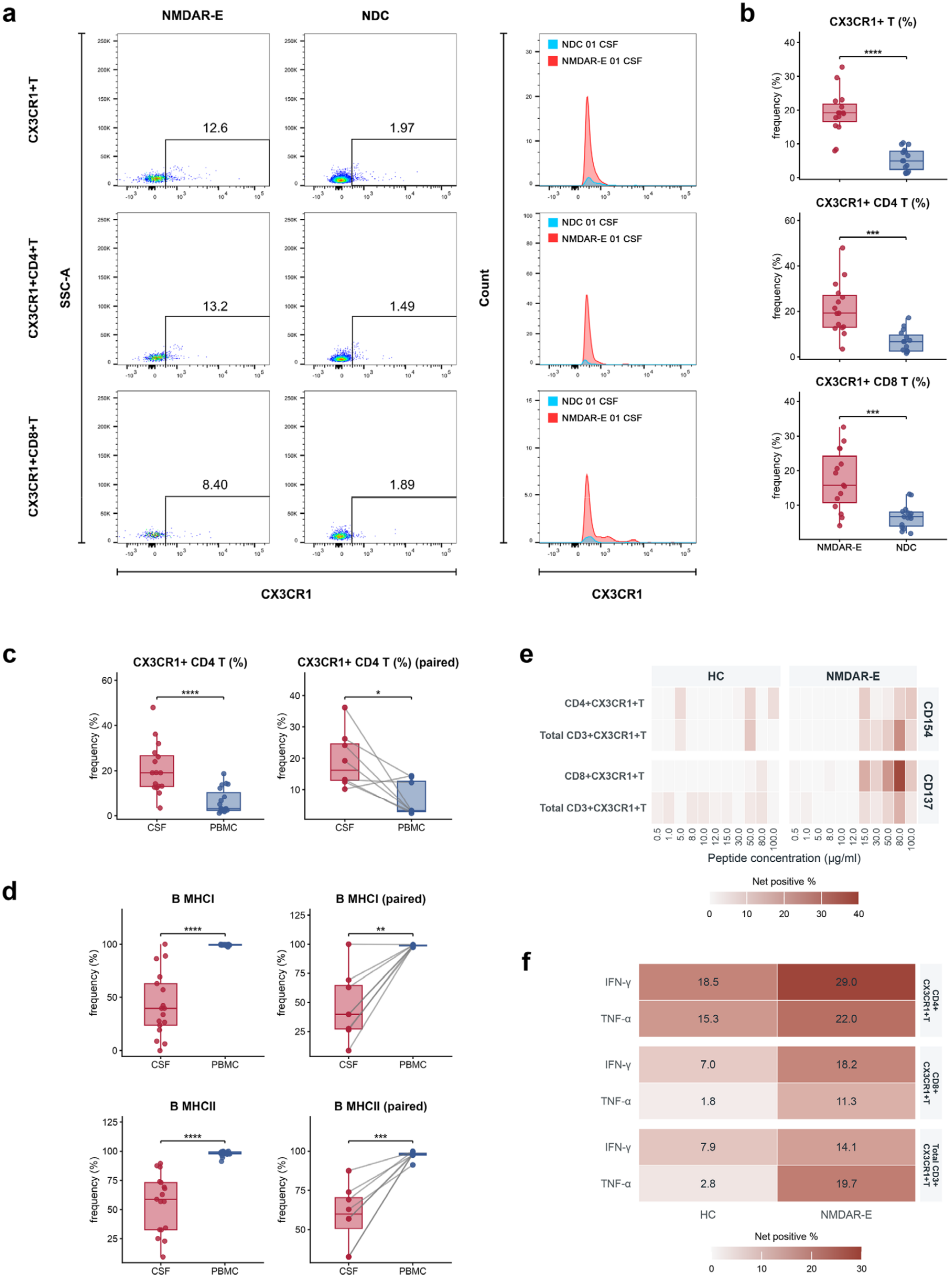
Flow‐cytometric validation of CX3CR1^+^ T cell expansion, B cell antigen‐presentation capacity and peptide‐induced functional responses in NMDAR‐E. (a) Representative flow‐cytometry plots and fluorescence‐intensity histograms showing total CX3CR1^+^ T cells and CX3CR1^+^ CD4^+^ and CX3CR1^+^ CD8^+^ T cell subsets in CSF from patients with NMDAR‐E and NDC. (b) Frequencies of total CX3CR1^+^ T cells and CX3CR1^+^ CD4^+^ and CX3CR1^+^ CD8^+^ T cell subsets in CSF from the NMDAR‐E and NDC groups. (c) Frequencies of CX3CR1^+^ CD4^+^ T cells in CSF and PBMC samples from patients with NMDAR‐E. Left, unpaired comparison between all available CSF and PBMC samples. Right, paired comparison of matched CSF and PBMC samples from the same patients. (d) Frequencies of MHC class I‐positive (MHC‐I^+^) and MHC class II‐positive (MHC‐II^+^) B cells in CSF and PBMC samples. Left, unpaired comparisons of CSF and PBMC samples; right, paired comparisons of matched CSF and PBMC samples from the same patients. (e) Heatmaps showing background‐corrected net‐positive frequencies of the activation‐induced markers (AIMs) CD154 and CD137 in total CX3CR1^+^ CD3^+^ T cells and the corresponding CD4^+^ and CD8^+^ subsets across different NR1 peptide concentrations in HCs and patients with NMDAR‐E. (f) Heatmaps showing background‐corrected net‐positive frequencies of intracellular IFN‐γ and TNF‐α in the corresponding CX3CR1^+^ T cell populations following stimulation with 80 µg/mL NR1 peptide.

To orthogonally validate the single‐cell findings, multiparameter flow cytometry was performed in an expanded independent cohort (clinical characteristics, Tables S3 and S4; flow cytometry gating strategy, Figure S9). Compared with neurological disease controls (NDCs), patients with NMDAR‐E exhibited significantly increased frequencies of total CX3CR1^+^ T cells as well as CX3CR1^+^ CD4^+^ and CX3CR1^+^ CD8^+^ T cell subsets in the CSF (Figure 7a,b). Paired analysis further demonstrated enrichment of CX3CR1^+^ CD4^+^ T cells in the CSF relative to matched peripheral blood (Figure 7c). Analysis of CX3CR1^+^ T cell differentiation subsets further revealed selective expansion of memory and effector populations in both the CD4^+^ and CD8^+^ compartments (Figure S9). In addition, the frequencies of MHC‐I^+^ and MHC‐II^+^ B cells were significantly lower in the CSF than in paired peripheral blood (Figure 7d). This compartmentalized pattern suggests that antigen presentation and T cell priming may occur mainly in the periphery, followed by recruitment or retention of activated CX3CR1^+^ T cells in the CNS.

We next stimulated PBMCs ex vivo with GluN1_356‐385_ peptide to evaluate the antigen peptide‐induced activation and effector responses of CX3CR1^+^ T cells (Figure S10). After background subtraction, patients with NMDAR‐E consistently exhibited higher frequencies of both CD154^+^ and CD137^+^ responder cells across multiple peptide concentrations than HCs (Figure 7e). ICS further demonstrated that CX3CR1^+^ T cells from patients produced higher levels of IFN‐γ and TNF‐α following peptide stimulation, with enhanced cytokine responses observed in the total CX3CR1^+^ ,CD4^+^ CX3CR1^+^, and CD8^+^ CX3CR1^+^ T cell subsets (Figure 7f). Collectively, these orthogonal validation and functional assays support CNS enrichment of CX3CR1^+^ T cells together with enhanced effector responses following NR1 peptide stimulation in NMDAR‐E.

## 3 Discussion

While prior investigations from our group and others have delineated the phenotypic landscape of B cell subsets and immunoglobulin class switching in NMDAR‐E, the multifaceted immunopathological cascades that drive disease initiation and progression remain to be fully elucidated [23, 24, 25, 26, 27]. In this study, integrative single‐cell multi‐omic profiling identifies clonally expanded CX3CR1^+^ T cells as a prominent T cell population potentially implicated in NMDAR‐E immunopathology, as peripherally activated CX3CR1^+^ CD4^+^ and CD8^+^ T cell subsets undergo clonal expansion and exhibit high predicted interaction scores between dominant TCR clonotypes and GluN1 derived epitopes spanning the N368/G369 region. In the periphery, these cells show enhanced crosstalk with activated naïve‐like B cells, an interaction that may promote adaptive immune activation and B cell effector functions, while concurrently displaying a broad activation program characterized by metabolic and proteolytic remodeling coupled with neuroinflammation‐associated transcriptional signatures. Importantly, enrichment of these cells in patient CSF supports their capacity to infiltrate the CNS compartment, a finding corroborated by multiparameter flow cytometry in an independent cohort, wherein ex vivo NR1 peptide stimulation reveals enhanced activation and elevated production of IFN‐γ and TNF‐α by patient‐derived CX3CR1^+^ T cells. These cells may contribute to ongoing neuroinflammation through mechanisms that may include antigen‐associated responses and antigen independent bystander activation, which, paired with their chemotactic profile particularly involving the CX3CL1–CX3CR1 axis, may facilitate recruitment to or retention within NMDAR‐rich regions such as the hippocampus and frontal cortex, where they could potentially influence neuron‐microglia homeostasis [28, 29]. These findings broaden current understanding of NMDAR‐E pathogenesis beyond B‐cell‐mediated autoantibody production, and identify CX3CR1^+^ T cells as a disease‐associated inflammatory effector population associated with peripheral T‐B cell collaboration and CNS‐compartmentalized injury, highlighting their potential value for immune stratification and motivating future investigation of more selective immunomodulatory strategies.

In autoimmune diseases of the CNS, the role of B cells as APCs has received growing research attention. While associations between HLA gene variants and NMDAR‐E susceptibility remain inconsistent across independent study cohorts [30, 31], accumulating evidence indicates that B cells from NMDAR‐E patients exhibit significant upregulation of DEGs related to both MHC class II and class I molecules, suggestive of activated antigen presentation pathways [17]. Transcriptomic data from the present study demonstrate activated BCR signaling and MHC‐related transcriptional programs in peripheral B cells, including elevated expression of HLA‐DRB5, and naïve‐like B cell subsets also exhibit strengthened intercellular communication with CX3CR1^+^ T cells, findings collectively supporting a functional role for peripheral B cells in promoting T cell activation. By contrast, paired flow cytometric analysis detects fewer MHC‐expressing B cells in the CSF relative to peripheral blood, a distribution pattern consistent with the presence of antigen‐experienced, antibody‐secreting B cells within the intrathecal compartment [32]. Given that CNS‐derived antigens can drain into cervical lymph nodes via CSF circulation and gain access to the peripheral immune system [33, 34], B cells may cooperate with other APCs to present these antigenic peptides and activate autoreactive T cells endowed with the capacity to traverse the blood‐brain barrier and enter the CNS [35, 36]. In this study, increased expression of antigen presentation‐associated molecules on peripheral blood B cells suggests that antigen exposure in cervical lymph nodes may enhance their capability to prime T cells in the periphery, and together these findings indicate that B cells contribute to NMDAR‐E pathogenesis not solely through autoantibody production, but also via antigen presentation and downstream promotion of T cell activation.

Autoreactive T cells recognize self‐derived peptides presented by MHC molecules through their TCRs, thereby initiating antigen‐specific immune response cascades [37, 38]. In the present cohort, peripheral CD4^+^ and CD8^+^ CX3CR1^+^ T cells from NMDAR‐E patients display activated TCR signaling, enriched immune‐related pathways, metabolic transcriptional alterations, and marked clonal expansion, with several dominant clonotypes exhibiting high predicted interaction scores for GluN1‐derived epitopes, findings suggestive of potential antigen‐associated clonal selection [39, 40, 41]. Ex vivo peptide stimulation assays further provide complementary functional evidence of NR1 peptide responsiveness, as NR1 peptide challenge upregulates CD137 and CD154 expression on CX3CR1^+^ T cells and induces robust production of IFN‐γ and TNF‐α. CD137 is induced on the surface of activated CD4^+^ and CD8^+^ T cells and serves as a widely used marker for identifying antigen‐responsive CD8^+^ T cells [42], whereas CD154 represents a more characteristic marker of activated CD4^+^ T cells and can deliver CD40‐dependent helper signals to B cells [43]. Together with the metabolic transcriptional alterations observed in these cells, these activation features suggest an immunometabolic component of the CX3CR1^+^ T cell effector state, consistent with the recognized role of metabolic remodeling in shaping T‐cell activation and effector differentiation [44]. Cell communication analysis further suggests reciprocal regulatory interactions between CX3CR1^+^ T cells and APCs, as APCs such as B cells can activate T cells through MHC‐associated signaling pathways [45], and in turn, MIF secreted by CX3CR1^+^ T cells may act via CD74 and CD44 to support the survival and activation of B cells and monocytes [46]. Transcriptomic and flow cytometric analyses consistently demonstrate increased CX3CR1^+^ T cells in the CSF, and enhanced CD99‐associated intercellular communication provides indirect evidence for a migration‐prone cellular phenotype [47, 48]. Taken together, these observations characterize CX3CR1^+^ T cells as inflammatory effectors associated with putative GluN1 recognition, interactions with APCs, proinflammatory cytokine production, and CNS inflammation in the context of NMDAR‐E.

Furthermore, NMDAR‐E predominantly affects the hippocampus and frontotemporal cortex [49], brain regions characterized by abundant expression of the NMDA receptor NR1 subunit [50, 51] and elevated levels of the neuron‐derived chemokine fractalkine (CX3CL1) [52], a molecular mediator essential for NMDA receptor regulation and activity‐dependent synaptic plasticity [53]. The cognate receptor CX3CR1 is broadly expressed across multiple immune cell subsets and is recognized as a definitive marker of T cell differentiation [54, 55], and while the pathogenic involvement of CX3CR1‐expressing immune cells has been well documented in neurological disorders such as multiple sclerosis [56, 57], their precise pathological roles in NMDAR‐E have yet to be fully delineated. In this study, CX3CR1^+^ T cells are found to be preferentially enriched in the CSF, and their activated TCR signaling, cytotoxic potential, and chemotactic capacity collectively suggest that these cells may accumulate in CX3CL1‐rich regions of the CNS and contribute to local inflammatory processes. Notably, neurons can upregulate MHC class I molecules and antigen‐processing genes in response to IFN‐γ stimulation or during neuroinflammatory conditions, thereby acquiring the capacity to present antigenic peptides to T lymphocytes [58]. Upon antigen recognition, cytotoxic T cells may contribute to neuronal and synaptic injury through MHC class I‐restricted cytotoxicity, Fas‐mediated apoptotic signaling, and phagocyte‐mediated synaptic stripping [59, 60, 61], though whether CX3CR1^+^ T cells directly mediate antigen‐specific neuronal damage in NMDAR‐E remains to be empirically determined [62]. Additionally, within the CNS parenchyma, CX3CR1 is predominantly localized to microglia, where it mediates fractalkine signaling critical for microglial homeostasis and functional diversity, and the infiltration of CX3CR1^+^ T cells may perturb this neuron‐microglia communication axis, thereby amplifying neuroinflammatory responses and downstream pathological processes [63].

Emerging immunotherapeutic strategies, including T cell‐targeted therapies [64, 65], cytokine blockade agents [66], and chemokine axis modulation [67], have demonstrated therapeutic promise in other autoimmune disorders of the CNS, providing a mechanistic rationale for extending this therapeutic framework to the management of NMDAR‐E. The results of this study highlight the disease‐associated inflammatory and migratory features of CX3CR1^+^ T cells and suggest that their activation, recruitment to the CNS compartment, and functional interactions with B cells may represent potential targets for therapeutic intervention. However, from a translational perspective, given the physiological roles of CX3CR1 in immune surveillance, leukocyte trafficking, and microglial homeostasis, the potential therapeutic implications of CX3CR1^+^ T cells may lie less in broadly targeting CX3CR1 itself than in selectively modulating disease‐associated effector programs within this population. In this context, cell‐specific modulation or targeted delivery strategies may improve therapeutic selectivity while minimizing interference with the physiological functions of CX3CR1. Functional co‐culture assays and relevant in vivo disease models, together with direct antigen specificity assays, will be required to determine whether selective modulation of disease‐associated interactions between CX3CR1^+^ T cells and B cells can attenuate inflammatory T cell activation and whether the predicted TCR interactions with GluN1 derived epitopes reflect bona fide antigen specificity. Although exploratory analyses in the present cohort did not identify significant associations between CX3CR1^+^ T cell frequencies and selected clinical parameters, longitudinal and multicenter studies will be required to determine whether dynamic changes in CX3CR1^+^ T cell signatures can track disease activity or treatment response, predict prognosis, and support individualized immune stratification.

## 4 Conclusion

This study identifies CX3CR1^+^ T cells as a prominent disease‐associated T cell population in NMDAR‐E, characterized by clonal expansion, enhanced responsiveness to NR1 peptide stimulation, proinflammatory effector functions, preferential enrichment in the CSF, and the potential capacity for functional cooperation with B lymphocytes. Collectively, these cellular features indicate that CX3CR1^+^ T cells participate in both peripheral adaptive immune activation and intrathecal neuroinflammatory processes. These findings provide novel mechanistic insights into NMDAR‐E immunopathology and characterize CX3CR1^+^ T cells as NR1 peptide‐responsive inflammatory effectors with potential value for immune stratification and future investigation of selective immunomodulatory strategies.

## 5 Methods

### 5.1 Participants and Ethical Approval

This study enrolled three patients newly diagnosed with NMDAR‐E and three age‐ and sex‐matched HC from the Department of Neurology at West China Hospital, Sichuan University, between June 2021 and July 2022. The study was conducted in accordance with the Declaration of Helsinki and relevant local regulations and received approval from the Medical Ethics Committee of West China Hospital, Sichuan University (**Ethical Approval No. 2020 1304)**. All participants, or their legal guardians, provided written informed consent prior to enrollment.

For the disease group, inclusion criteria were: (1) age between 18 and 40 years; and (2) a confirmed diagnosis of NMDAR‐E based on the diagnostic criteria for autoimmune encephalitis [1] and the Chinese expert consensus on the diagnosis and management of autoimmune encephalitis (2022 edition) [68]. Exclusion criteria included: (1) concomitant malignancy, severe liver or kidney dysfunction, other neurological or autoimmune diseases, or infectious encephalitis; (2) prior immunosuppressive treatment before sample collection; and (3) the presence of additional autoimmune encephalitis or paraneoplastic antibodies in the CSF or serum. HCs were required to be 18–45 years of age, without a history of neurological disorders or recent acute infections, and confirmed healthy based on routine clinical assessments, including blood, urine, and stool tests, inflammatory markers, metabolic panels, brain imaging, and autoimmune encephalitis antibody testing.

To validate key findings, an independent locally generated scRNA‐seq cohort was analyzed, comprising four PBMC and three CSF samples from patients with NMDAR‐E and two PBMC samples from HCs. Because CSF samples from healthy individuals were unavailable, processed gene‐expression matrices for five CSF samples from individuals with IIH were retrieved from the Gene Expression Omnibus dataset GSE138266 and included as NDCs. The locally generated samples and the publicly available CSF control data were analyzed using harmonized downstream procedures for normalization, integration, dimensionality reduction, clustering, and cell‐type annotation. Validation analyses were conducted separately in the peripheral blood and CSF compartments.

Multiparameter flow cytometry was performed in a separate independent cohort. The peripheral blood cohort included 19 patients with NMDAR‐E and 11 HCs. The CSF cohort comprised 16 patients with NMDAR‐E and 15 NDCs. The NDC group included 10 patients with epilepsy or other seizure disorders, three with hydrocephalus or intracranial hypertension, one with vertigo and normal CSF findings, and one with visual impairment and normal CSF findings. An additional three patients with NMDAR‐E and three HCs, all of whom met the corresponding eligibility criteria described above, were enrolled in the ex vivo NR1 peptide stimulation assays. Detailed demographic and clinical characteristics are provided in Tables S3–S5.

### 5.2 Preparation of Single‐Cell Suspensions

Peripheral blood (5 mL per participant) was collected into ethylenediaminetetraacetic acid (EDTA) anticoagulant tubes. Blood samples were diluted 1:1 with 1× calcium‐ and magnesium‐free Dulbecco's phosphate‐buffered saline (D‐PBS) and carefully layered over an equal volume of Ficoll Paque in 50‐mL centrifuge tubes. The samples were centrifuged at 500 g for 20 min at 20°C using a horizontal rotor with the brake off. The interface layer containing PBMCs was harvested and transferred to 15‐mL tubes. Cells were washed twice with 10 mL of 1× D‐PBS at 300 g for 10 min per wash to remove residual plasma and platelets. After the final wash, PBMCs were resuspended in 1 mL of Roswell Park Memorial Institute (RPMI) 1640 medium supplemented with 0.04% bovine serum albumin (BSA; Corning, Cat. No. 100040CVR). Cell concentration, viability, clumping rate, and debris levels were assessed using a Luna cell counter and light microscopy. Only suspensions with >90% viability, <5% clumping, and low debris were used for downstream processing.

### 5.3 Single‐Cell Multi‐Omics Library Preparation

Single‐cell suspensions were adjusted to 700–1200 cells/µL. For CITE‐seq analysis, cells were stained with oligonucleotide‐conjugated TotalSeq‐C antibodies according to the manufacturer's instructions and washed to remove unbound antibodies. The stained cell suspensions were loaded onto the Chromium Next GEM Single Cell 5’ platform (10x Genomics) for generation of gel beads in emulsion.

Within each gel bead in emulsion, individual cells were lysed, and polyadenylated mRNA molecules were captured by barcoded oligo‐dT primers. During reverse transcription, cell barcodes and unique molecular identifiers (UMIs) were incorporated into the resulting cDNA, and template switching introduced a common sequence at the 5’ end of the cDNA. Following emulsion breakage, barcoded cDNA and antibody‐derived oligonucleotide tags were recovered and amplified.

Separate libraries were constructed for 5’ gene expression, TCR, and BCR enrichment, and antibody‐derived tags (ADT) according to the manufacturer's protocols. The resulting libraries were sequenced on an Illumina NovaSeq 6000 platform using paired‐end 150‐bp reads.

### 5.4 Data Preprocessing

Raw sequencing data were processed using the Cell Ranger software (v7.1.0). Reads were aligned to the human reference genome (hg38), and individual cells were identified based on unique cellular barcodes and UMIs. Cell Ranger generated a gene expression matrix along with counts for V (D) J sequences and, when applicable, surface protein expression. Following this initial processing, further quality control was performed to remove doublets and low‐quality cells. Cells were retained for downstream analysis if they met thresholds for the number of detected genes, UMI counts, log_10_ (genes per UMI), and exhibited low proportions of mitochondrial or hemoglobin gene expression. For multimodal data, ADT expression was integrated with RNA features using weighted nearest neighbor (WNN) analysis in Seurat, enabling joint clustering and dimensionality reduction across modalities.

### 5.5 Cell Clustering and Type Identification

The filtered expression matrices were imported into R and converted into Seurat objects using the CreateSeuratObject function in Seurat. Gene‐expression data were normalized using the NormalizeData function with the LogNormalize method, whereby gene counts in each cell were normalized to the total cellular counts, multiplied by a scale factor, and log transformed. Highly variable genes were identified using the FindVariableFeatures function. Batch effects across samples were addressed using canonical correlation analysis through the FindIntegrationAnchors and IntegrateData functions. The integrated expression data were subsequently centered and scaled using the ScaleData function before principal component analysis (PCA).

Dimensionality reduction was carried out using PCA on the top variable genes, and the number of significant principal components was determined by inspecting elbow plots. Nonlinear dimensionality reduction and visualization were performed using UMAP via the RunUMAP function. For CITE‐seq data, ADT count matrices were generated from the sequencing reads using CITE‐seq‐Count (v1.4.3) and imported into Seurat as a separate ADT assay. ADT counts were normalized using the centered log‐ratio transformation, centered and scaled, and subjected to PCA. RNA and ADT modalities were then integrated by constructing a weighted nearest‐neighbor graph for joint clustering and dimensionality reduction.

Cell clusters were annotated by comparing expression profiles using the FindAllMarkers function in Seurat to identify marker genes. Annotations were refined by cross‐referencing well‐established markers from the literature, the CellMarker2.0 database, and additional insights from the ACT single‐cell annotation platform [69]. Further subclustering was performed on individual cell types using the subset function and reapplying the clustering pipeline.

### 5.6 Gene set Variation and Enrichment Analyses

GSVA was used to obtain exploratory pathway‐activity scores from log‐normalized single‐cell expression data. For the discovery‐cohort analyses, KEGG and GOBP gene sets were obtained from the Molecular Signatures Database (MSigDB, v7.2) and imported using GSEABase (v1.44.0). GSVA (v1.30.0) was applied with default parameters to calculate an activity score for each pathway in each cell. For the multi‐subpopulation analyses shown in Figures 2d and 3d,i, cells were stratified by annotated subpopulation and clinical condition. For each subpopulation‐condition stratum, pathway scores were compared with those of all remaining strata using limma (v3.38.3). Moderated t statistics were used for heatmap visualization. Pathways with a nominal *p* value < 0.05 were ranked by their t statistics, after which representative pathways were selected for visualization on the basis of their biological relevance. For analyses restricted to predefined CX3CR1‐expressing T cell populations, direct comparisons between disease and control cells were performed using the same per‐cell GSVA‐limma framework.

GSEA was performed separately within each prespecified cell population to identify biological processes and pathways differentially enriched between patients with NMDAR‐E and the corresponding control group. HCs were used for peripheral‐blood comparisons, whereas individuals with IIH were used as controls for the validation cerebrospinal‐fluid comparison. Raw UMI counts from cells belonging to each population were aggregated by donor to generate pseudobulk count matrices. Lowly expressed genes were removed using filterByExpr (min.count = 5 and min.total.count = 10) in edgeR (v4.8.2). Library sizes were normalized using the trimmed mean of M‐values method, and group differences were estimated using a quasi‐likelihood negative‐binomial model with robust dispersion estimation. Genes were ranked in descending order by sign log_2_ fold change (log_2_FC) × √F, where F denotes the quasi‐likelihood F statistic. Negligible deterministic offsets based on the log_2_FC and gene identity were added to resolve exact ties without materially altering the ranking. Preranked GSEA was performed using fgseaMultilevel in the fgsea package (v1.36.0) against three complete gene‐set libraries: the MSigDB Hallmark collection (v2024.1.Hs; 50 gene sets), human KEGG pathways (KEGG Release 114.0; 359 pathways), and the MSigDB C5 GOBP (v2024.1.Hs; 7,608 gene sets). After intersection with the ranked gene list, gene sets containing 15–500 genes were retained. GSEA was performed using standard weighting (scoreType = “std”), with nPermSimple = 10 000 and eps = 0. All eligible pathways in each complete library were tested before pathways were selected for visualization. For each comparison, nominal *p* values were adjusted separately across all eligible pathways within each complete gene‐set library using the Benjamini‐Hochberg method. Library‐wise false‐discovery rates (padj_library) below 0.05 were considered statistically significant and were used for pathway selection and reporting. Positive normalized enrichment scores indicate enrichment in NMDAR‐E, whereas negative scores indicate enrichment in the corresponding control group. For cell‐subset analyses, the primary analysis included all donors contributing at least one target cell. Sensitivity analyses were repeated after requiring each donor to contribute at least 5 or 10 target cells.

### 5.7 Cell‐Cell Communication Analysis

To elucidate intercellular communication networks, we applied the CellChat R package (v1.1.3). The normalized expression matrix was imported to create a CellChat object with the createCellChat function. Data were preprocessed using the identifyOverExpressedGenes and identifyOverExpressedInteractions functions, followed by projection with the projectData function. Communication probabilities were computed using computeCommunProb and refined with the filterCommunication (min.cells = 10) and computeCommunProbPathway functions. The final cell‐cell communication network was aggregated with the aggregateNet function to reveal ligand‐receptor interactions that may underlie disease pathogenesis.

### 5.8 Immune Repertoire Sequencing and Analysis

TCR and BCR libraries were generated using the 10x Genomics Single Cell V(D)J Enrichment Kits and sequenced on the Illumina NovaSeq 6000 platform with PE150 reads. Raw V(D)J data were processed using Cell Ranger (v7.1.0), and productive receptor sequences and clonotypes were assigned to individual cells. For TCR analysis, clonotypes were defined according to Cell Ranger clonotype IDs and their corresponding CDR3 amino acid sequence combinations. Clonal expansion was quantified by the number of cells assigned to each clonotype.

TCR repertoire diversity was evaluated separately for CD4^+^ and CD8^+^ T cells at the biological‐sample level. The primary analysis included all cells with valid TCR assignments, whereas paired TRA‐TRB cells were analyzed as a sensitivity set. Diversity was assessed using Shannon entropy, the inverse Simpson index, Pielou evenness, clonality, and the Gini index.

### 5.9 Flow Cytometric Immunophenotyping

PBMC and CSF cells were analyzed by multiparameter flow cytometry. Cells were stained with fluorochrome‐conjugated antibodies against CD45, CD3, CD4, CD8, CD19, CCR7, CD45RA, CX3CR1, HLA class I, and HLA class II. Doublets were excluded using forward scatter area (FSC‐A) vs. forward scatter height (FSC‐H), followed by identification of lymphocytes and CD3^+^ T cells. CD4^+^ and CD8^+^ T cells were further classified as Tn (CCR7^+^ CD45RA^+^), Tcm (CCR7^+^ CD45RA^−^), Tem (CCR7^−^ CD45RA^−^), and terminally differentiated effector memory RA‐positive T cell (Temra) (CCR7^−^ CD45RA^+^) subsets. The frequencies of CX3CR1^+^ cells were quantified within total CD3^+^ T cells, CD4^+^ and CD8^+^ T cells, and the corresponding differentiation subsets. Within the CD3^−^ population, CD19^+^ B cells were identified, and the frequencies of HLA class I‐positive and HLA class II‐positive B cells were quantified. Data were acquired using a BD FACSLyric flow cytometer and analyzed with FlowJo (v10.9.0). Representative gating strategies are shown in Figure S9.

### 5.10 PBMC Stimulation and AIM/ICS Flow Cytometry

T cell responses were evaluated by activation‐induced marker (AIM) analysis and intracellular cytokine staining (ICS). PBMCs were isolated from venous blood, washed with PBS, counted, and resuspended in RPMI 1640 medium supplemented with 5% human AB serum. Cells were seeded at 1 × 10^6^ cells in 1 mL per well in 12‐well plates and rested for 4 h at 37°C with 5% CO_2_. For AIM analysis, PBMCs were then exposed to the commercially synthesized GluN1_356‐385_ peptide (MedChemExpress, HY‐P5912; purity, 99.95%) at the indicated concentrations for 12 h. For ICS analysis, the peptide was used at a final concentration of 80 µg/mL. For each sample, a matched peptide free culture was processed in parallel and used as the negative control to define baseline activation, rather than an unrelated control peptide, whereas HKCA (1 × 10^7^ particles/mL) was included as a positive antigen control.

To reduce CD154 internalization, an anti‐CD40 antibody was added at 1 µg/mL at the beginning of peptide exposure. After 12 h of culture, BD Pharmingen Leukocyte Activation Cocktail with BD GolgiPlug (catalog no. 550583; 2 µL/mL), containing phorbol 12‐myristate 13‐acetate, ionomycin, and brefeldin A, was added to all experimental conditions. Cells were then incubated for an additional 4–6 h before staining.

T cell responses were evaluated by AIM and ICS. Surface staining included CD3, CD4, CD8, CX3CR1, CD69, CD137, and CD154. After fixation and permeabilization, intracellular IFN‐γ and TNF‐α were detected. Samples were acquired using a BD FACSymphony A5 SE flow cytometer and analyzed with FlowJo (v10.9.0). AIM and cytokine responses were quantified as the frequencies of marker positive cells within the corresponding CX3CR1^+^ T cell populations. Peptide induced responses were background corrected for each sample by subtracting the corresponding frequency measured in the matched peptide free control. Representative gating strategies are shown in Figure S10.

### 5.11 Statistical Analysis

Statistical analyses were performed in R. For the identification of cluster‐specific marker genes, cell‐level comparisons were conducted using the Wilcoxon rank‐sum test implemented in Seurat. These analyses were used for cell‐type annotation.

For differential expression analysis between clinical groups, the biological sample was treated as the independent statistical unit. Within each cell type or subcluster, raw UMI counts were summed across cells from the same participant to generate sample‐level pseudobulk expression matrices. Genes with low expression across samples were excluded before analysis. Differential expression between the NMDAR‐E and HC groups was assessed using edgeR, with the disease group included in the design matrix. P values were adjusted for multiple comparisons using the Benjamini‐Hochberg method, and genes with an adjusted *p* value below 0.05 were considered statistically significant.

Unless otherwise specified, all statistical tests were two‐sided. Data visualization, including UMAP plots, violin plots, dot plots, and heatmaps, was performed using Seurat and ggplot2.

## Author Contributions

L.Y. (**Lin Yan**): supervised the project, guided the study design, and contributed to manuscript writing. E.Z. (**Erdi Zhang**): performed core bioinformatic analyses and visualization, and prepared manuscript figures. Y.M. (**Yuwen Ma**): wrote the first draft and contributed to revisions; assisted in bioinformatic analyses. Z.M. (**Zirui Meng**): collected and prepared samples and acquired data. S.L. (**Sisi Li**): contributed to sample collection and experimental validation. Y.R. (**Yimeng Ren**): assisted in bioinformatic analyses, focusing on interpretation and functional annotation. X.T. (**Xiaohe Tian**): provided imaging support and contributed to data analysis and manuscript revision. Y.H. (**Yangyi He**): assisted in clinical sample collection and provided clinical data support. G.B. (**Giuseppe Battaglia**): contributed to study design and data interpretation, and revised the manuscript. D.Z. (**Dong Zhou**): provided clinical supervision and contributed to data interpretation. Y.L. (**Yun Liao**): provided guidance on flow cytometry and related experiments, contributed to experimental design and data interpretation, and revised the manuscript. B.Y. (**Binwu Ying**): supervised experiments and data analysis, and revised the manuscript. J.L. (**Jinmei Li**): supervised the neurological aspects and contributed to manuscript revision. M.W. (**Minjin Wang**): coordinated sample and data collection, contributed to study design, and co‐wrote the manuscript.

## Funding

This work was supported by the National Natural Science Foundation of China (NSFC, 32401233), the 1·3·5 Project for Disciplines of Excellence from West China Hospital of Sichuan University (ZYGD23036), and the “Qimingxing” Research Fund for Young Talents (HXQMX0089).

## Conflicts of Interest

The authors declare no conflicts of interest.

## Supporting information




**Supporting File 1**: advs77608‐sup‐0001‐SuppMat.pdf.

**Supporting File 2**: advs77608‐sup‐0002‐SuppMat.xlsx.

## Data Availability

The public CSF control data are available in the Gene Expression Omnibus (GEO) under accession GSE 138266. The data generated in this study are available from the corresponding author upon reasonable request.
