# Supplementary material for: Single‐Cell Profiling Reveals Clonally Expanded CX3CR1+ T Cells in Anti‐NMDA Receptor Encephalitis
Source: Adv Sci (Weinh). 2026 Sep 8:e77608. Online ahead of print. doi: 10.1002/advs.77608 (PMC13554385; doi:10.1002/advs.77608)
Supplement: Supplementary file 1 [file ADVS-9999-e77608-s001.pdf]

Supplementary Information

Single-Cell Profiling Reveals Clonally Expanded CX3CR1^{+} T Cells in Anti-NMDA Receptor Encephalitis

Lin Yan[1,2,3]†, Erdi Zhang[1,2,3]†, Yuwen Ma[1]†, Zirui Meng[4], Sisi Li[5], Yimeng Ren[6], Xiaohe Tian[7]，Yangyi He[8], Giuseppe Battaglia[9,10], Dong Zhou[11], Yun Liao[1,2,3], Binwu Ying[1,2,3,12]*, Jinmei Li[11]* & Minjin Wang[1,2,3,11]**

E-mail: Yun Liao(liaoy@scu.edu.cn), Binwu Ying (yingbinwu@scu.edu.cn), Jinmei Li (lijinmei@wchscu.cn) and Minjin Wang (wang.minjin@outlook.com).

L. Yan, E. Zhang, Y. Ma, Y. Liao, B. Ying, M. Wang

Department of Laboratory Medicine, West China Hospital, Sichuan University, Chengdu 610041, Sichuan, China

L. Yan, E. Zhang, Y. Liao, B. Ying, M. Wang

Clinical Laboratory Medicine Research Center, West China Hospital, Sichuan University, Chengdu 610041, Sichuan, China

L. Yan, E. Zhang, Y. Liao, B. Ying, M. Wang

Sichuan Clinical Research Center for Laboratory Medicine, Chengdu 610041, Sichuan, China

Z. Meng

Department of Laboratory Medicine, West China Hospital of Stomatology, Sichuan University, Chengdu 610041, Sichuan, China

S. Li

Department of Breast Cancer Center, Chongqing University Cancer Hospital, Chongqing 400030, China

Y. Ren

LKS Faculty of Medicine, The University of Hong Kong, Hong Kong, China

X. Tian
Functional and Molecular Imaging Key Laboratory of Sichuan Province, Huaxi MR Research Centre (HMRRC), Department of Radiology, West China Hospital, Sichuan University, Chengdu 610000, Sichuan, China

Y. He
Department of Surgery and Surgical Specializations, University of Barcelona, Barcelona 08036, Spain

G. Battaglia
Institute for Bioengineering of Catalunya (IBEC), The Barcelona Institute of Science and Technology, Barcelona 08028, Spain

G. Battaglia
Department of Chemistry, University College London (UCL), London WC1H 0AJ, UK

D. Zhou, J. Li, M. Wang
Department of Neurology, West China Hospital, Sichuan University, Chengdu 610041, Sichuan, China

B. Ying
Institution of Medical and Engineering Integration for Molecular Diagnosis, West China Hospital, Sichuan University, Chengdu 610041, Sichuan, China

This file includes the following Supplementary Figures and legends. Supporting Tables are provided in **Supplementary_Tables.xlsx**.

1. Supporting Figures

Figure S1. Canonical marker gene expression for major PBMC cell types and subgroup composition of NK cells and monocytes in the discovery cohort.

Figure S2. Additional characterization of B cell subsets in the discovery cohort.

Figure S3. Additional characterization of $CD8^+$ T cell subsets in the discovery cohort.

Figure S4. Additional characterization of $CD4^+$ T cell subsets in the discovery cohort.

Figure S5. Functional characterization of $CX3CR1^+$ T cells in the discovery cohort.

Figure S6. TCR repertoire diversity and clonal expansion characteristics of $CD4^+$ and $CD8^+$ T cell subsets in the discovery cohort.

Figure S7. Differential expression and pathway analyses of $CX3CR1^+$ T cells in the validation cohort.

Figure S8. Molecular characteristics of highly expanded T cells and B-cell clonotype distributions in the discovery cohort.

Figure S9. Gating strategy and quantification of $CX3CR1^+$ T cell subsets in the flow cytometry cohort.

Figure S10. Gating strategy for ex vivo functional validation by flow cytometry.

2. Supporting Tables are supplied as .xlsx files.

1. Supplementary Figures

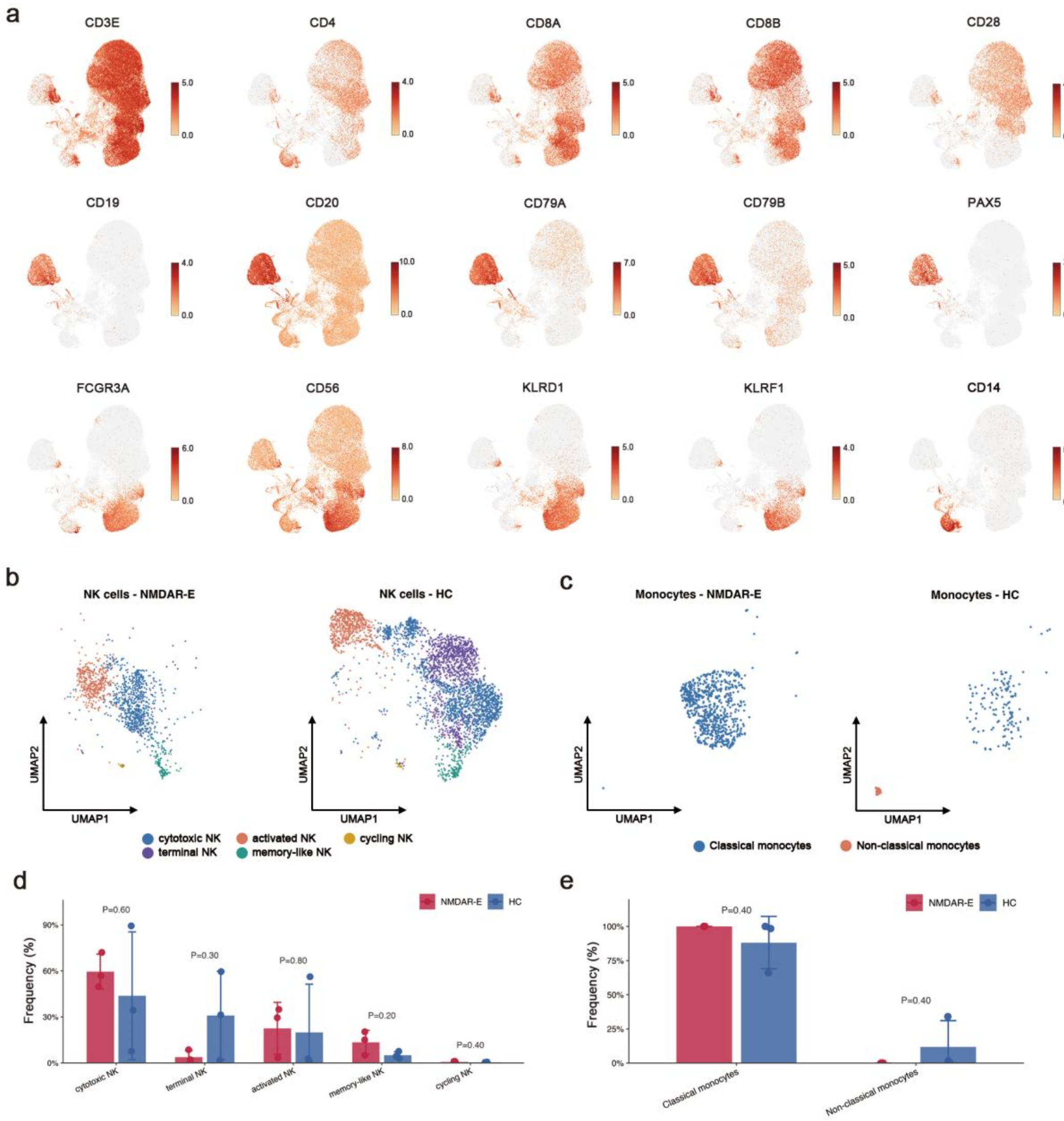

Figure S1 | Canonical marker gene expression for major PBMC cell types and subgroup composition of NK cells and monocytes in the discovery cohort.

a, UMAP plots showing transcriptomic expression of canonical marker genes used for major immune cell type annotation in PBMCs from the discovery cohort, including T cell markers (CD3E, CD4, CD8A, CD8B, and CD28), B cell markers (CD19, CD20, CD79A, CD79B, and PAX5), NK cell markers (FCGR3A, CD56, KLRD1, and KLRF1), and the monocyte marker CD14.

b, UMAP projections showing NK cell subpopulations in the NMDAR-E (left) and HC (right) groups, including cytotoxic NK cells, terminal NK cells, activated NK cells, memory-like NK cells, and cycling NK cells.

c, UMAP projections showing monocyte subpopulations in the NMDAR-E (left) and HC (right) groups, including classical and non-classical monocytes.

d, Relative frequencies of NK cell subpopulations in the NMDAR-E and HC groups.

e, Relative frequencies of monocyte subpopulations in the NMDAR-E and HC groups. Each dot represents one participant (NMDAR-E, n = 3; HC, n = 3), and bars indicate the mean ± SD. Between-group comparisons were performed using a two-sided Welch’s t-test. P values were adjusted for multiple comparisons within each panel using the Benjamini–Hochberg method.

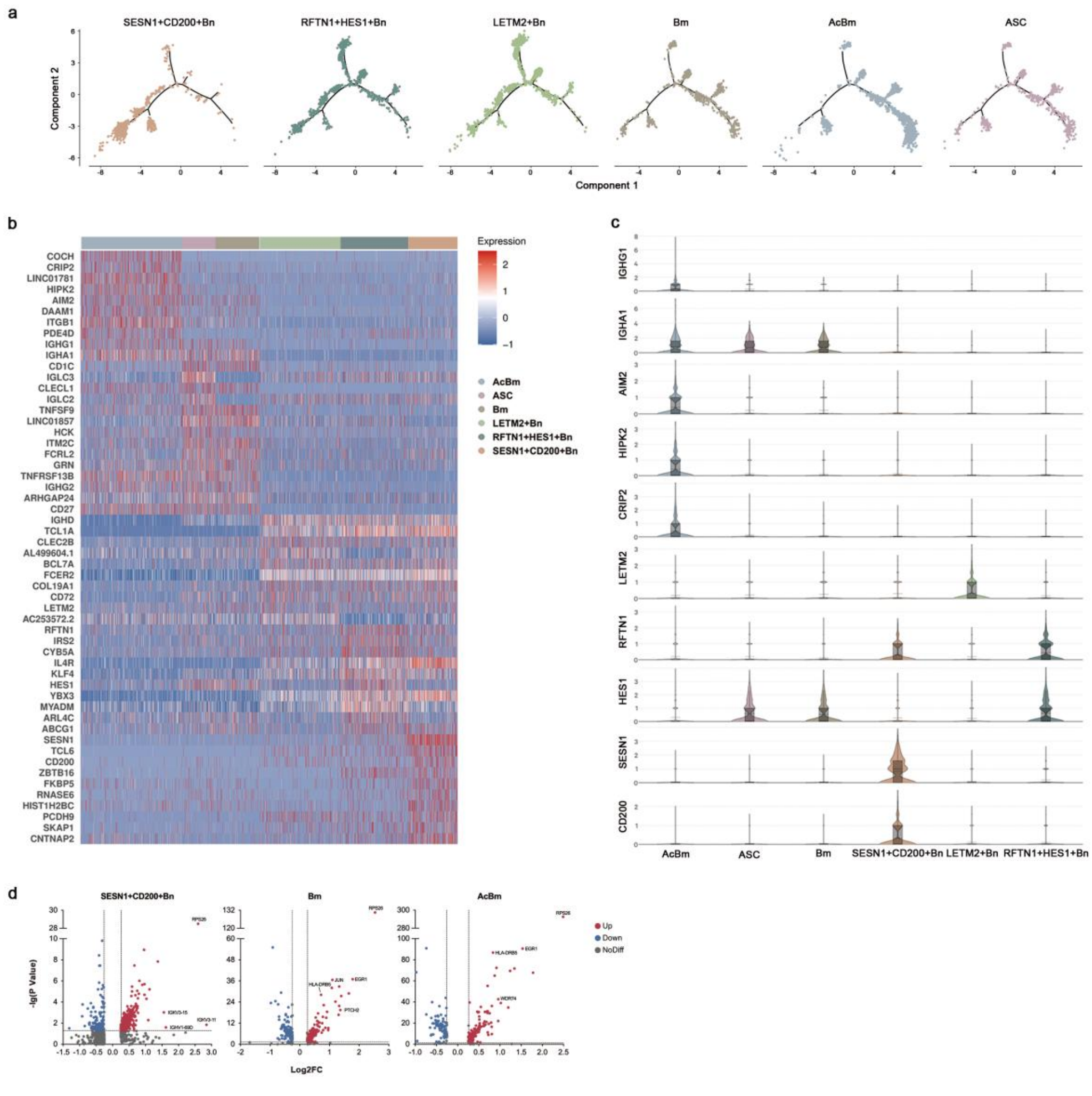

Figure S2 | Additional characterization of B cell subsets in the discovery cohort.

a, Pseudotime trajectories of the six annotated B cell subsets inferred by Monocle2.

b, Heatmap showing the top 10 marker genes for each annotated B cell subset. Marker

genes were ranked by gene diff, defined as the ratio of the proportion of cells expressing the gene in the corresponding subset (PCT1) to that in all remaining subsets (PCT2).

c, Violin plots showing the expression of representative marker genes across the annotated B cell subsets.

d, Volcano plots showing differentially expressed genes (DEGs) between NMDAR-E and HC in the $SESN1^+$ $CD200^+$ Bn, Bm, and AcBm subsets. Representative upregulated genes are highlighted.

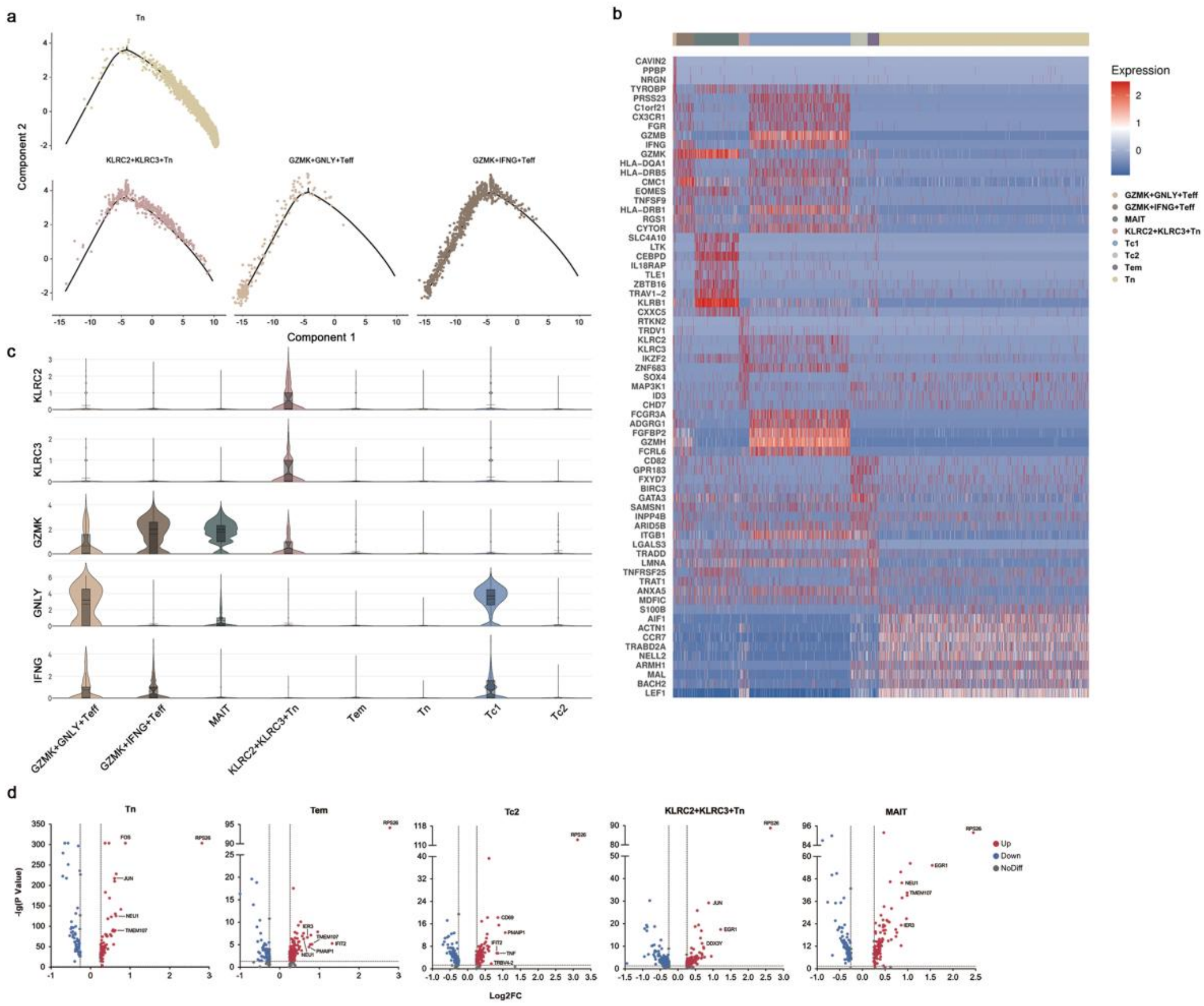

Figure S3 | Additional characterization of $CD8^+$ T cell subsets in the discovery cohort.

a, Pseudotime trajectories of selected $CD8^+$ T cell subsets inferred by Monocle2.

b, Heatmap showing the top 10 marker genes for each annotated $CD8^+$ T cell subset. Marker genes were ranked by gene diff, defined as the ratio of the proportion of cells expressing the gene in the corresponding subset (PCT1) to that in all remaining

subsets (PCT2).

c, Violin plots showing the expression of representative marker genes across the annotated CD8⁺ T cell subsets.

d, Volcano plots showing DEGs between NMDAR-E and HC in the Tn, Tem, Tc2, KLRC2⁺ KLRC3⁺ Tn, and MAIT subsets. Representative upregulated genes are highlighted.

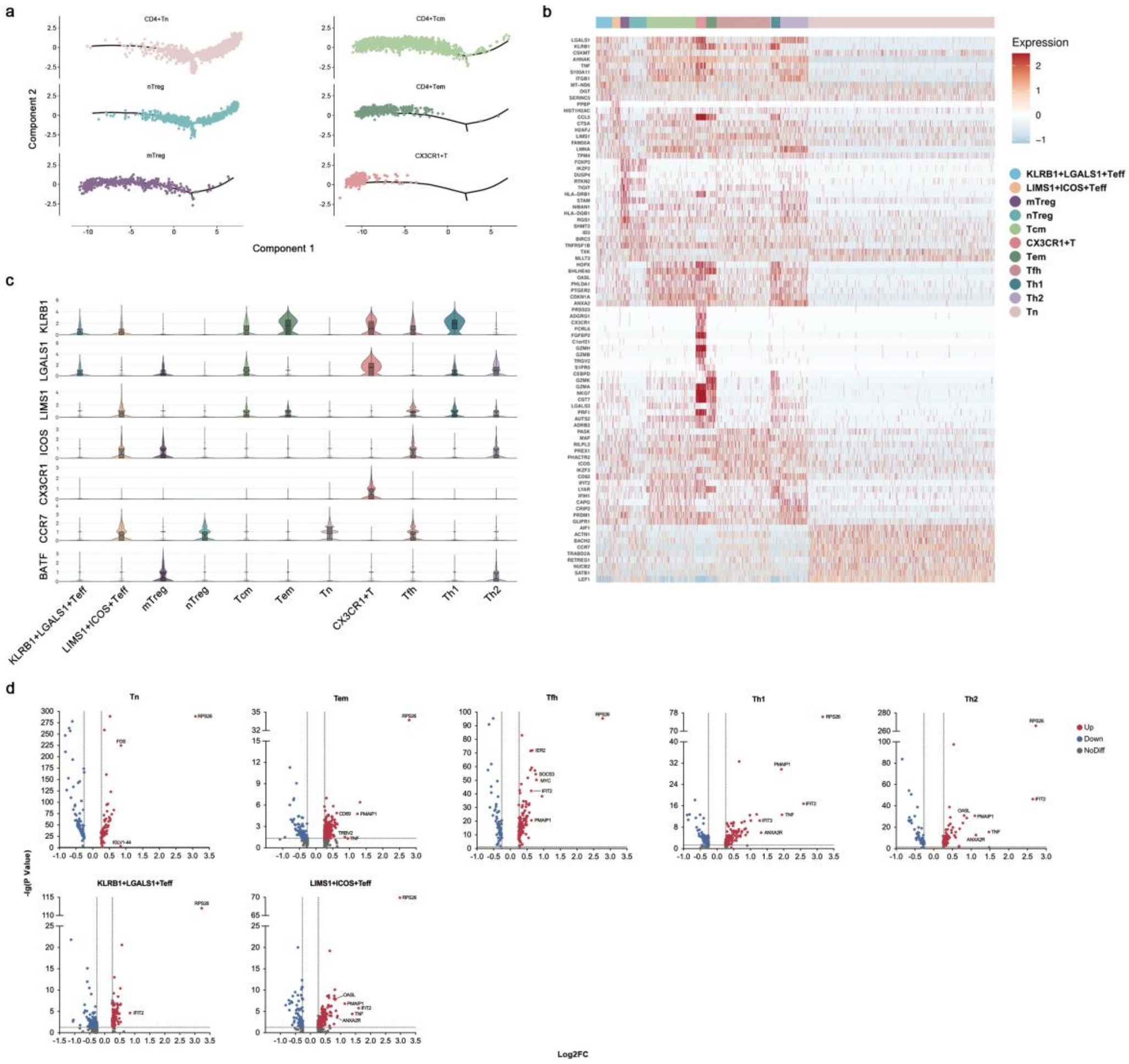

Figure S4 | Additional characterization of CD4⁺ T cell subsets in the discovery cohort.

a, Pseudotime trajectories of selected CD4⁺ T cell subsets inferred by Monocle2.

b, Heatmap showing the top 10 marker genes for each annotated CD4⁺ T cell subset. Marker genes were ranked by gene diff, defined as the ratio of the proportion of cells expressing the gene in the corresponding subset (PCT1) to that in all remaining subsets (PCT2).

c, Violin plots showing the expression of representative marker genes across the annotated CD4^{+} T cell subsets.

d, Volcano plots showing DEGs between NMDAR-E and HC in the Tn, Tem, Tfh, Th1, Th2, KLRB1^{+} LGALS1^{+} Teff, and LIMS1^{+} ICOS^{+} Teff subsets. Representative upregulated genes are highlighted.

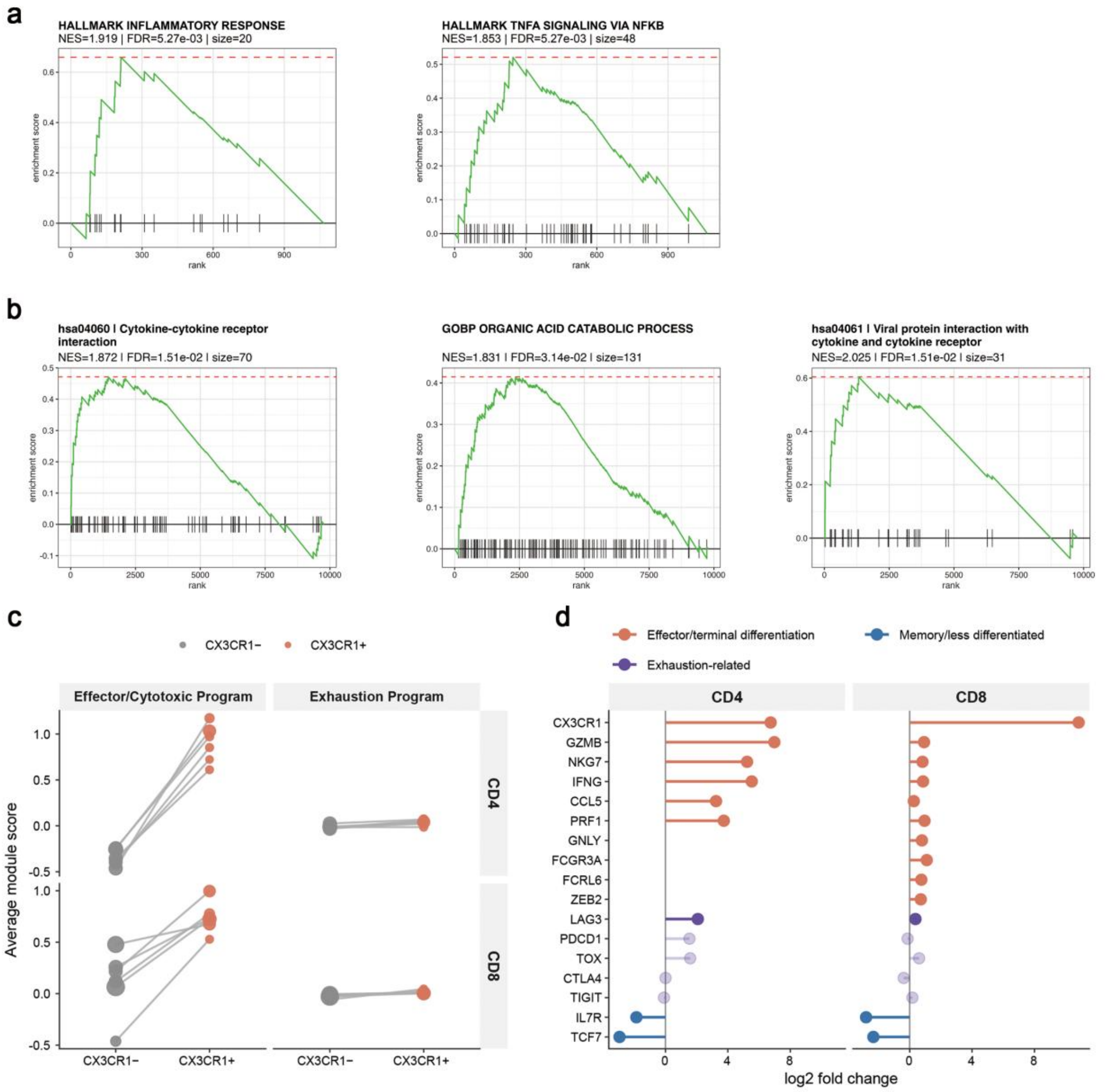

Figure S5 | Functional characterization of CX3CR1^{+} T cells in the discovery cohort.

a, Representative GSEA plots showing enrichment of the Hallmark Inflammatory Response and Hallmark TNFA Signaling via NF-κB gene sets in CD4^{+} CX3CR1^{+} T cells.

b,Representative GSEA plots showing enrichment of KEGG cytokine − cytokine

receptor interaction, GOBP organic acid catabolic process, and KEGG viral protein interaction with cytokine and cytokine receptor pathways in $CD8^{+}$ $CX3CR1^{+}$ T cells.

c, Comparison of average effector/cytotoxic and exhaustion module scores between $CX3CR1^{-}$ and $CX3CR1^{+}$ cells within the $CD4^{+}$ and $CD8^{+}$ T cell compartments. Module scores were calculated using Seurat AddModuleScore and averaged at the sample level for each CX3CR1 group. The effector/cytotoxic module included IFNG, TNF, GZMB, GZMK, PRF1, NKG7, and CCL5 for $CD4^{+}$ T cells, with GNLY, CCL4, and CST7 additionally included for $CD8^{+}$ T cells. The exhaustion module included PDCD1, LAG3, TIGIT, HAVCR2, CTLA4, TOX, TOX2, BATF, PRDM1, ENTPD1, and CXCL13 in both compartments. Lines connect $CX3CR1^{-}$ and $CX3CR1^{+}$ cells from the same sample. Point size represents the number of cells contributing to each sample-level average.

d, Log2 fold changes in representative genes associated with effector/terminal differentiation, exhaustion-related, and memory/less differentiated programs in $CX3CR1^{+}$ relative to $CX3CR1^{-}$ $CD4^{+}$ and $CD8^{+}$ T cells. Differential expression was assessed using a sample-paired pseudobulk DESeq2 model. Positive values indicate higher expression in $CX3CR1^{+}$ T cells.

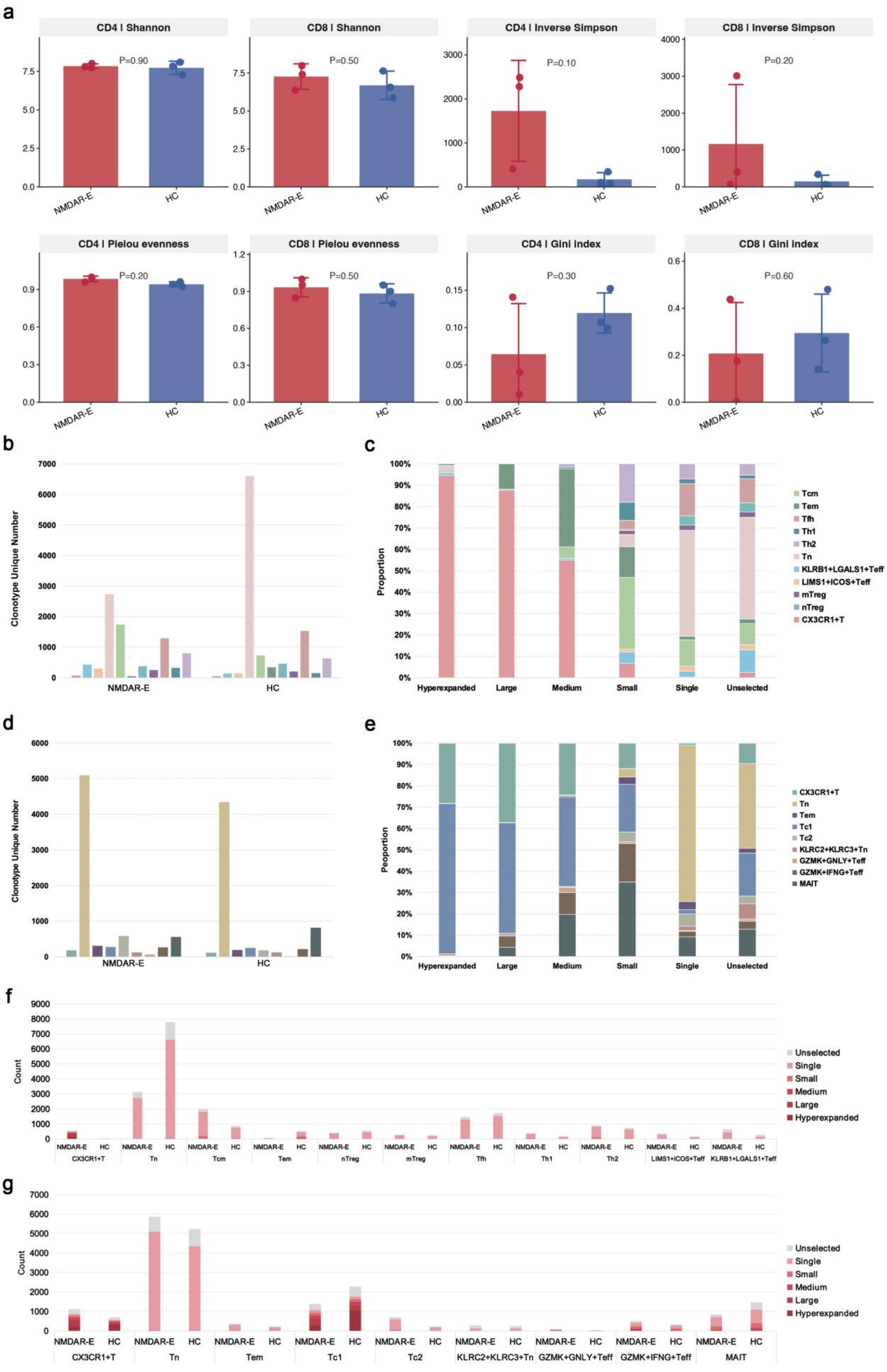

Figure S6 | TCR repertoire diversity and clonal expansion characteristics of

$CD4^+$ and $CD8^+$ T cell subsets in the discovery cohort.

a, Comparison of TCR repertoire diversity between the NMDAR-E and HC groups. Shannon diversity, inverse Simpson index, Pielou evenness, and Gini index were calculated separately for $CD4^+$ and $CD8^+$ T cells at the participant level. Each dot represents one biological sample (NMDAR-E, n = 3; HC, n = 3), and bars indicate the mean ± SD. Between-group comparisons were performed using two-sided Welch's t-tests. P values were adjusted for multiple comparisons across the four diversity metrics within each T-cell lineage using the Benjamini–Hochberg method.

b, Numbers of unique clonotypes detected in each $CD4^+$ T cell subset in the NMDAR-E and HC groups.

c, Relative composition of $CD4^+$ T cell subsets across different clonal expansion categories (hyperexpanded, large, medium, small, single, and unselected).

d, Numbers of unique clonotypes detected in each $CD8^+$ T cell subset in the NMDAR-E and HC groups.

e, Relative composition of $CD8^+$ T cell subsets across different clonal expansion categories (hyperexpanded, large, medium, small, single, and unselected).

f, Numbers of cells belonging to different clonal expansion categories within each $CD4^+$ T cell subset in the NMDAR-E and HC groups.

g, Numbers of cells belonging to different clonal expansion categories within each $CD8^+$ T cell subset in the NMDAR-E and HC groups.

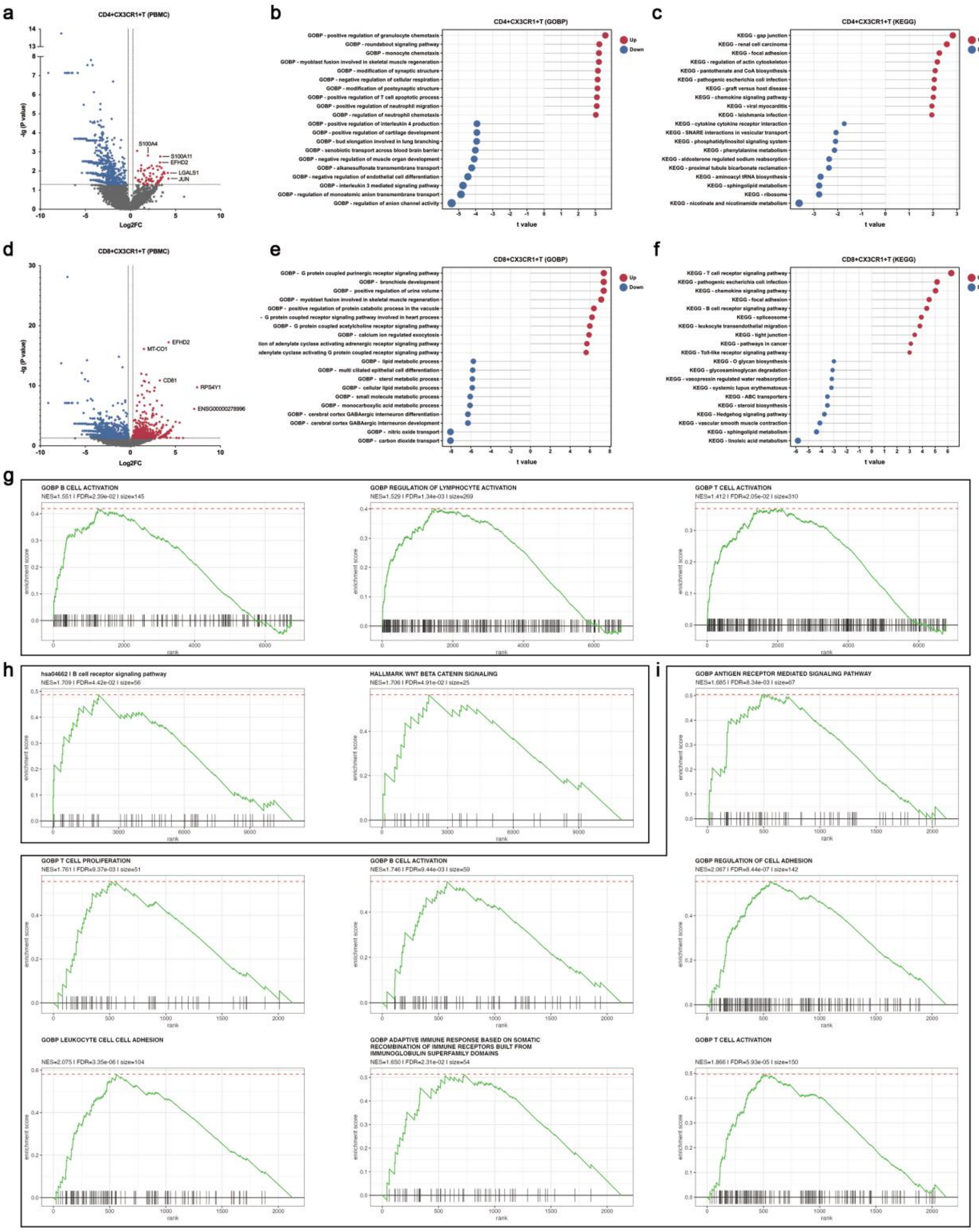

Figure S7 | Differential expression and pathway analyses of CX3CR1^{+} T cells in the validation cohort.

a, Volcano plot showing DEGs between NMDAR-E and HC in PBMC-derived CD4^{+} CX3CR1^{+} T cells. Representative differentially expressed genes are labeled.

b-c, GSVA of PBMC-derived CD4^{+} CX3CR1^{+} T cells showing representative significantly altered GOBP (b) and KEGG (c) pathways.

d, Volcano plot showing DEGs between NMDAR-E and HC in PBMC-derived CD8^{+}

CX3CR1⁺ T cells. Representative differentially expressed genes are labeled.

e-f, GSVA of PBMC-derived CD8⁺ CX3CR1⁺ T cells showing representative significantly altered GOBP (e) and KEGG (f) pathways.

g, Representative gene set enrichment analysis (GSEA) plots of PBMC-derived CD4⁺ CX3CR1⁺ T cells from the validation cohort.

h, Representative GSEA plots of PBMC-derived CD8⁺ CX3CR1⁺ T cells from the validation cohort.

i, Representative GSEA plots of CSF-derived CX3CR1⁺ T cells from the validation cohort.

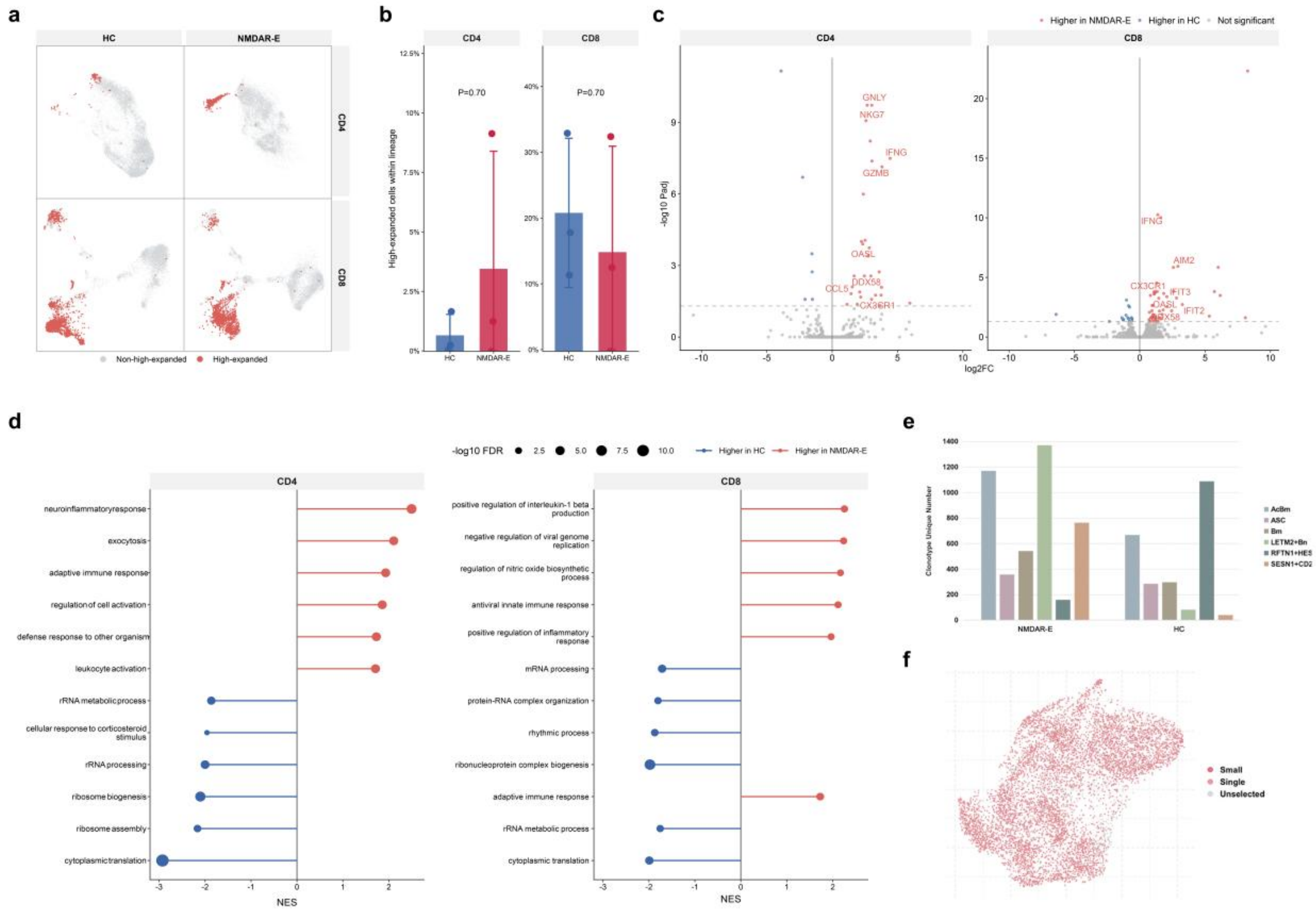

Figure S8 | Molecular characteristics of highly expanded T cells and B-cell clonotype distributions in the discovery cohort.

a, UMAP projections of CD4⁺ and CD8⁺ T cells from the discovery cohort, showing the distribution of highly expanded clonotypes (clone size ≥ 5) and clonotypes that were not highly expanded(clone size < 5) in the HC and NMDAR-E groups.

b, Comparison of the proportions of highly expanded T cells within the CD4⁺ and CD8⁺ T-cell lineages between the NMDAR-E and HC groups. For each participant,

the proportion was calculated as the number of T cells assigned to clonotypes with a clone size ≥ 5 divided by the total number of T cells with valid TCR assignments within the corresponding lineage. Each dot represents one biological sample (NMDAR-E, n = 3; HC, n = 3), and bars indicate the mean ± SD. Between-group comparisons were performed using two-sided Welch's t-tests.

c, Volcano plots showing DEGs between High-expanded T cells from the NMDAR-E and HC groups in CD4+ (left) and CD8+ (right) T cells. Representative differentially expressed genes are labeled.

d, Gene set enrichment analysis (GSEA) of High-expanded CD4+ (left) and CD8+ (right) T cells comparing the NMDAR-E and HC groups. Representative significantly enriched biological processes are shown. Positive normalized enrichment scores (NES) indicate pathways enriched in NMDAR-E, whereas negative NES indicate pathways enriched in HC. Dot size represents −log10(FDR).

e, Numbers of unique clonotypes detected in each B-cell subset in the NMDAR-E and HC groups.

f, UMAP of B cells colored according to clonal expansion categories (Small, Single, and Unselected).

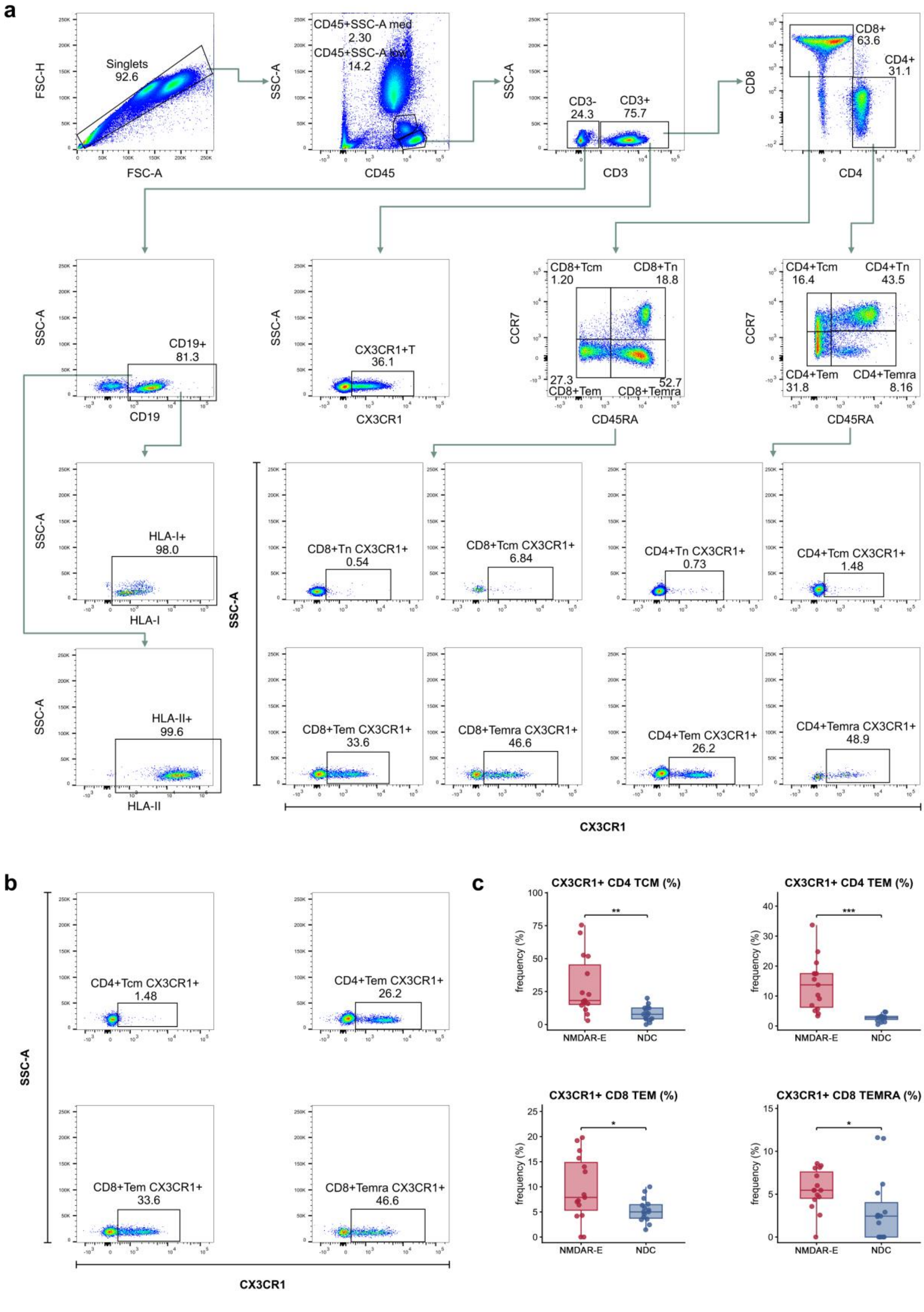

Figure S9 | Gating strategy and quantification of CX3CR1^{+} T cell subsets in the flow cytometry cohort.

a, Representative flow cytometry gating strategy used for immunophenotyping in the independent flow cytometry cohort. After exclusion of doublets, CD45^{+} SSC-A^{low}

leukocytes were gated and separated into $CD3^+$ T cells and $CD3^-$ cells. Within $CD3^+$ T cells, $CX3CR1^+$ cells were quantified in total T cells and in $CD4^+$ and $CD8^+$ T cell populations. $CD4^+$ and $CD8^+$ T cells were further subdivided into naïve (Tn), central memory (Tcm), effector memory (Tem) and terminally differentiated effector memory RA-positive (Temra) subsets according to CCR7 and CD45RA expression, followed by quantification of CX3CR1 expression within each subset. $CD3^-$ cells were gated as $CD19^+$ B cells, followed by analysis of HLA-I and HLA-II expression.

b, Enlarged representative terminal gates for $CX3CR1^+$ $CD4^+$ Tcm, $CX3CR1^+$ $CD4^+$ Tem, $CX3CR1^+$ $CD8^+$ Tem and $CX3CR1^+$ $CD8^+$ Temra cells.

c, Frequencies of the indicated $CX3CR1^+$ T-cell subsets in CSF samples from patients with NMDAR-E and neurological disease controls (NDCs). Each point represents one participant (NMDAR-E, n = 16; NDC, n = 15). Boxes indicate the median and interquartile range, and whiskers extend to 1.5 times the interquartile range. Between-group comparisons were performed using two-sided Mann–Whitney U tests. P values were adjusted across the four subset comparisons using the Benjamini–Hochberg method. *$P < 0.05$, **$P < 0.01$, and ***$P < 0.001$.

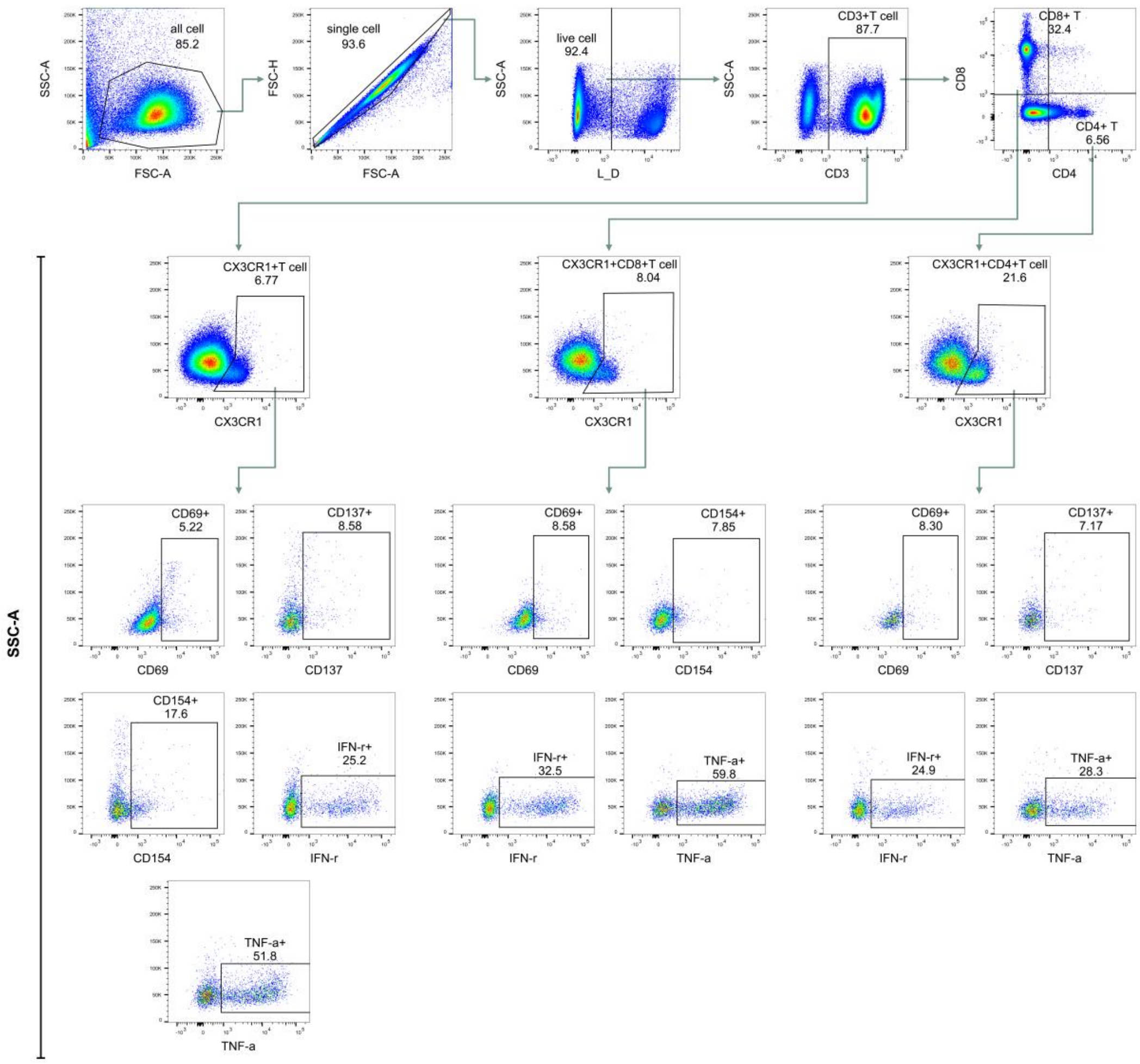

Figure S10 | Gating strategy for ex vivo functional validation by flow cytometry. Representative flow cytometry gating strategy used to assess T cell activation and cytokine production following in vitro stimulation of PBMCs with the GluN1$_{356\text{-}385}$ peptide. PBMCs were sequentially gated on all cells, singlets, live cells, CD3$^+$ T cells, and CD4$^+$ or CD8$^+$ T cells. CX3CR1$^+$ cells were subsequently identified within total CD3$^+$ T cells, CD4$^+$ T cells, and CD8$^+$ T cells. Activation-induced markers (AIM), including CD69, CD137, and CD154, as well as intracellular cytokines (IFN-γ and TNF-α), were analyzed in the corresponding CX3CR1$^+$ T cell populations.

2. Supplementary Table

Supplementary Table 1. Characteristics of NMDAR-E patients and control individuals for single-cell RNA sequencing.

Supplementary Table 2. Clinical characteristics of subjects in the discovery and validation cohorts.

Supplementary Table 3. Demographic and clinical characteristics of participants in the peripheral blood flow cytometry cohort.

Supplementary Table 4. Demographic and clinical characteristics of participants in the cerebrospinal fluid flow cytometry cohort.

Supplementary Table 5. Clinical characteristics of subjects included in the ex vivo GluN1 peptide stimulation assays.

Supplementary Table 6. Differentially expressed genes between NMDAR-E and the corresponding control groups in both the discovery and validation cohorts.

Supplementary Table 7. GSVA results comparing NMDAR-E and controls in the discovery and validation cohorts.
